# Ecological Momentary Assessment of Self-Harm Thoughts and Behaviors: Systematic Review of Constructs From the Integrated Motivational-Volitional Model

**DOI:** 10.2196/63132

**Published:** 2024-12-09

**Authors:** Lizzy Winstone, Jon Heron, Ann John, Olivia J Kirtley, Paul Moran, Jennifer Muehlenkamp, Rory C O'Connor, Becky Mars

**Affiliations:** 1 Population Health Sciences University of Bristol Bristol United Kingdom; 2 Swansea University Medical School Swansea University Swansea United Kingdom; 3 Center for Contextual Psychiatry KU Leuven Leuven Belgium; 4 NIHR Biomedical Research Centre at the University Hospitals Bristol NHS Foundation Trust Bristol United Kingdom; 5 University of Wisconsin Eau Claire Eau Claire, WI United States; 6 Suicidal Behaviour Research Lab School of Health and Wellbeing University of Glasgow Glasgow United Kingdom

**Keywords:** integrated motivational-volitional model, IMV model, ecological momentary assessment, suicidal and nonsuicidal self-harm thoughts and behaviors

## Abstract

**Background:**

The integrated motivational-volitional model (IMV) is one of the leading theoretical models of suicidal thoughts and behavior. There has been a recent proliferation in the assessment of suicidal and nonsuicidal self-harm thoughts and behaviors (SHTBs) in daily life.

**Objective:**

This systematic review synthesized evidence from ecological momentary assessment (EMA) studies in the SHTB literature to address the following questions: (1) Which constructs in the IMV model have been assessed using EMA, and how have they been assessed? (2) Do different constructs from the IMV model fluctuate in daily life? (3) What is the relationship between the different IMV constructs and SHTBs in daily life?

**Methods:**

Consistent with the PRISMA (Preferred Reporting Items for Systematic Reviews and Meta-Analyses) guidelines, we conducted systematic searches of 5 databases—Web of Science, Embase, MEDLINE, PsycINFO, and Europe PMC Preprints—from inception to March 26, 2024.

**Results:**

Our searches resulted in the inclusion and narrative synthesis of 53 studies across 58 papers. A total of 15 IMV constructs were measured using EMA across the included papers. The most frequently measured constructs were thwarted belongingness (24/58, 41% of the papers), future thinking (20/58, 34% of the papers), and perceived burdensomeness (16/58, 28% of the papers). The least frequently measured constructs were humiliation, social problem-solving, mental imagery, and perceived capability for suicide. None of the included papers measured memory biases, goals, norms, or resilience using EMA. Comparison of intraclass correlation coefficients (45/58, 78% of the papers) revealed moderate but inconsistent within-person variance across all the examined constructs. We found evidence (39/58, 67% of the papers) of concurrent associations between almost all constructs and SHTBs in daily life, with some evidence that entrapment, shame, rumination, thwarted belongingness, hopelessness, social support, and impulsivity are additionally associated with SHTBs in lagged (ie, longitudinal) relationships.

**Conclusions:**

Comparisons were hindered by variation in methodology, including the populations studied, EMA sampling scheme, operationalization of IMV constructs and SHTBs, and statistical approach used. Our findings suggest that EMA studies are a useful methodology for examining risk factors for SHTBs; however, more research is needed for some IMV constructs. Quality assessment suggested several areas for improvement in the reporting of EMA studies in this field.

**Trial Registration:**

PROSPERO CRD42022349514; https://www.crd.york.ac.uk/prospero/display_record.php?RecordID=349514

## Introduction

### Background

Suicidal and nonsuicidal self-harm thoughts and behaviors (SHTBs) are a global public health concern, with estimates suggesting that >14.6 million individuals are affected by self-harm (defined as any form of deliberate self-injury irrespective of motivation or intent) each year and >700,000 deaths per year are attributable to suicide [1]. Understanding factors that contribute to the development of SHTBs is essential for prevention and early intervention.

**Figure 1:**
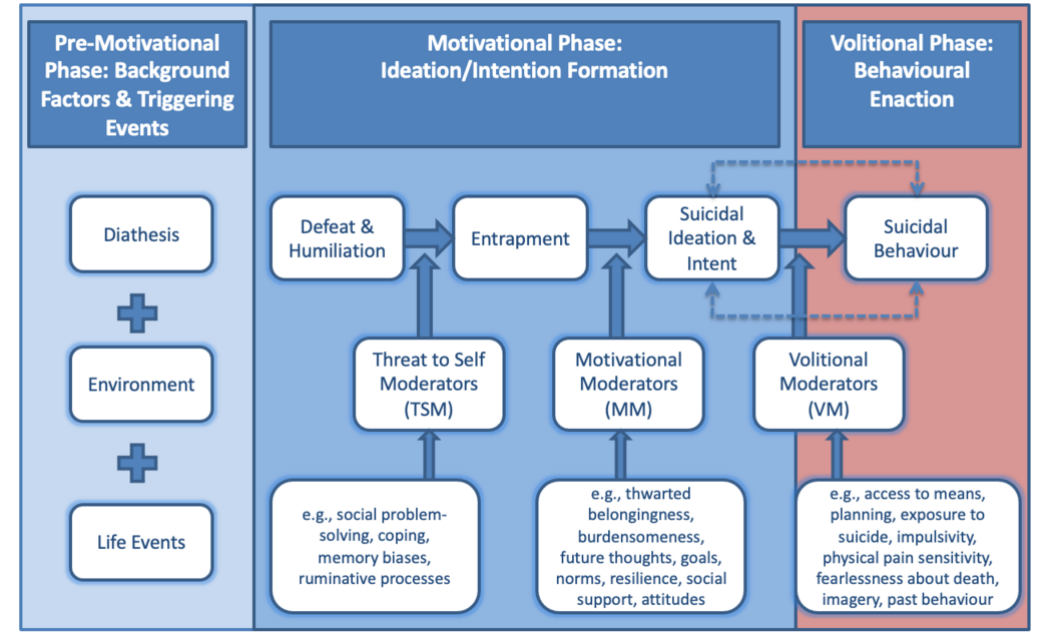
The integrated motivational-volitional model of suicidal behavior (adapted with permission from O’Connor and Kirtley [3]). MM: motivational moderators; TSM: threat-to-self moderators; VM: volitional moderators.

The integrated motivational-volitional model (IMV) is one of the leading theoretical models of suicidal behavior, developed by O’Connor [2] and later refined by O’Connor and Kirtley [3] (Figure 1 [3]). The IMV model consists of 3 phases: the premotivational stage, describing the biopsychosocial context in which suicidal thoughts and behavior may emerge; the motivational phase, describing the factors that lead to the development of suicidal thoughts; and the volitional phase, describing the factors that predict the transition from thoughts to behaviors [3].

Core constructs of the motivational phase include feelings of defeat, humiliation, and entrapment, which drive the emergence of suicidal thoughts. These can be facilitated or impeded by the presence of moderating variables, termed “threat to self,” and “motivational” moderators. The transition from suicidal thoughts to behaviors is, in turn, influenced by the presence of “volitional moderators” [2,3]. Although the IMV model was developed in relation to suicidal thoughts and behavior, the central concepts of the model can also be applied to nonsuicidal SHTBs [4].

A recent systematic review of studies testing the IMV model of suicidal behavior yielded support for the central components of the model (ie, the defeat-entrapment–suicidal ideation [SI] pathway)—but called for more focus on the constructs referred to as threat-to-self and motivational moderators in the model [5]. The review identified extensive testing of the model using cross-sectional retrospective methods while highlighting the need for more prospective (including intensive longitudinal) testing of IMV constructs [5].

### Ecological Momentary Assessment

Recent technological advances have made it easier for researchers to gain insights into SHTBs in real time using intensive longitudinal methods [6-8]. These methods are commonly referred to in the literature as experience sampling methods (ESMs), ambulatory assessment, daily diaries, and ecological momentary assessment (EMA). From this point onward, for brevity, we use the term *EMA* to refer to this methodology. EMA is a diary-based method involving repeated and frequent assessment of feelings, behaviors, and contexts in an individual’s natural environment. This minimizes recall bias, maximizes ecological validity, and enables dynamic processes to be captured. Assessment may be once daily (daily diaries); repeated throughout the day at pseudorandomized or specific times in a signal-contingent sampling scheme; or repeated based on reporting of a specific event, such as an act of nonsuicidal self-injury (NSSI; event contingent) [6]. Despite concerns regarding the demands placed on research participants from intensive sampling, it has been found to be both acceptable and feasible, with generally good compliance reported [9]. While there have been further concerns about the repeated reporting of mental states having an influence on an individual’s mental state, there is no strong evidence of such iatrogenic effects [7,8].

Existing EMA studies of suicidal thoughts have shown them to be highly variable over time [10,11]; however, less is known about the extent to which proximal risk factors for SHTBs, such as those proposed by the IMV model, fluctuate in daily life. Understanding the dynamic nature of risk factors (within-person variability) and their moderators is essential to tailoring interventions and risk assessments. It is important to understand whether risk factors and moderators are better characterized by individual differences (between-person variability) or momentary changes in experiences (within-person variability) [11,12]. Examining intraclass correlation coefficients (ICCs—see the Data Analysis section for more details) enables distinction between trait-level risk factors (high ICC and high between-person variability)—supporting longer-term strategies for intervention—and state-level risk factors (low ICC and high within-person variability)—for which acute, timely, and situation-specific intervention may be more appropriate [11]. A recent proliferation of EMA studies in the field of suicide and self-harm has prompted the need for a comprehensive synthesis of this literature. While others have reviewed EMA literature on self-harm [13-15], suicidal thoughts [9,13,16,17], and interpersonal processes in an SHTB context [18], EMA studies specifically assessing key constructs across both the motivational and volitional phases of the IMV model have not yet been synthesized. In addition, existing reviews of EMA studies have typically focused on the relationship between risk factors and SHTBs, and less attention has been paid to the characteristics of the risk factors themselves.

We conducted a systematic review of the SHTB literature in which constructs from the motivational and volitional phases of the IMV model were assessed using EMA. We offer a narrative synthesis, describing how IMV constructs were assessed in daily life, characterizing their within-person variability, and summarizing the evidence of the proximal relationships between each IMV construct and SHTBs. We identified gaps in the evidence base and proposed directions for future research.

### Primary Review Questions

The review questions are as follows:

1. Which of the key constructs in the IMV model have been assessed in EMA studies—in the context of suicidal or nonsuicidal SHTBs—and how have they been assessed?
2. Do different constructs from the IMV model show fluctuation in daily life when measured in the context of suicidal or nonsuicidal SHTBs, and what is their within-person variability?

### Secondary Review Question

The secondary review question is as follows:

1. What is the relationship between the different IMV constructs and suicidal and nonsuicidal SHTBs in daily life?

## Methods

### Overview

This review was preregistered on the PROSPERO database (CRD42022349514) and on the Open Science Framework (OSF) [19]. The PRISMA (Preferred Reporting Items for Systematic Reviews and Meta-Analyses) guidelines were followed (Multimedia Appendix 1), in addition to the Non-Intervention, Reproducible, and Open Systematic Review guidelines [20]. We searched the databases Web of Science, Embase, MEDLINE, PsycINFO, and Europe PMC Preprints. We also hand searched reference lists and citations of the included papers for additional papers not returned by the database searches.

The full search terms and strategy are available on the OSF [19]. We searched for studies that used intensive longitudinal methods, often referred to as EMA, experience sampling, ambulatory assessment, or daily diary methods. We did not establish a date limit on the search. The initial search was conducted in October 2022, yielding 40 papers, with an updated search in November 2023 yielding an additional 13 papers. A final presubmission updated search conducted in March 2024 yielded an additional 6 studies over 5 papers.

Records were exported to, stored, and managed using the application Rayyan (Rayyan Systems Inc). In total, 2 (blinded) authors independently screened the papers for inclusion based on the titles and abstracts simultaneously against the inclusion and exclusion criteria, with disagreements resolved through discussion. One author conducted full screening of the selected papers based on the full text. Full details are available in the corresponding OSF project page [19].

### Inclusion and Exclusion Criteria

We included studies that had assessed at least one of the IMV constructs in daily life using intensive longitudinal data collection methods (ie, EMA). These factors include the core motivational phase factors (defeat, shame, humiliation, and entrapment), threat-to-self moderators (problem-solving, coping, memory bias, and rumination), motivational moderators (thwarted belongingness, burdensomeness, future thinking, goals, norms, resilience, social support, and attitudes), and volitional moderators (suicide planning, exposure to self-harm, impulsiveness, pain sensitivity, fearlessness about death, and imagery). To be included, the studies needed to report details of within-person variability in IMV constructs (ie, ICC; see the Data Analysis section). Where not reported, we contacted the authors to request this information. If ICCs were not available and no association between at least one IMV construct and SHTBs was reported, the study was excluded.

We included PhD theses published on the internet and excluded studies that were meta-analyses, reviews, editorials, or commentaries, as well as articles not written in English. We did not limit the inclusion of studies according to population or participant characteristics, and both clinical and nonclinical samples were included.

We will not describe the assessment of SHTBs in this study as this has been covered by previous reviews [8,14,16,17].

### Data Analysis

Data extraction (template available on the OSF [19]) included a range of descriptive data for each study, including demographic information about the sample, whether the study sampled a clinical or community-based population, study design, and information about the IMV constructs measured. ICCs were extracted to describe the level of within-person variability in each construct. ICCs indicate the proportion of a variable’s variance that is due to between- and within-person variability [21]. The within-person variability is calculated as 1 minus the ICC. When within-person variability is low, this means that the variability in a construct is mostly due to differences between people and there is little fluctuation in the construct in people (ie, the construct may be considered more traitlike than statelike). For example, a hypothetical ICC of 0.83 would indicate just 17% within-person variance, suggesting that the construct is more traitlike and shows little fluctuation in people. Conversely, an ICC of 0.26 would indicate 74% within-person variance, suggesting that the construct is more statelike and shows large fluctuation in people over time.

Where studies tested associations between IMV constructs and SHTBs—either concurrent or lagged—these associations were also extracted. Quality assessment of the reporting of the studies was conducted according to an EMA-specific quality assessment tool [18]. Example reporting criteria included participant training in the EMA protocol being detailed in the Methods section, justification of the sample size, compliance rate and reasons for noncompliance, discussion of EMA-specific limitations, and open code for analysis (the full criteria are available on the OSF [19]).

## Results

### Description of the Included Studies

**Figure 2:**
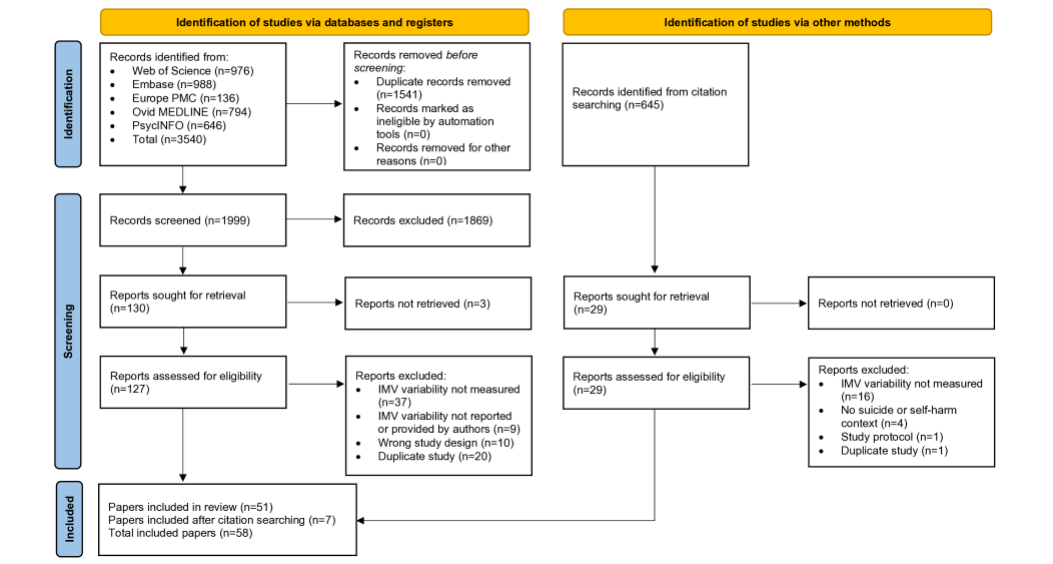
PRISMA (Preferred Reporting Items for Systematic Reviews and Meta-Analyses) flow diagram. IMV: integrated motivational-volitional model.

A total of 53 studies (unique samples) were included in this review (Figure 2 provides the PRISMA flowchart) across 58 papers, all from higher-income countries, with most from North America (n=40, 75%), the United Kingdom (n=3, 6%), and Germany (n=3, 6%). The studies varied substantially in terms of population, sample size, design, and constructs measured. Several papers (11/58, 19%) reported different analyses using the same study sample (ie, the same sample was used to report different IMV constructs across different papers). To avoid double counting of samples and designs, Table 1 reports a summary of 53 studies (58 papers reporting results from 53 independent samples).

**Table 1:** Overview of the samples of the included studies (n=53).

		Values
**Sample size**		
	Total (combined), N	4523^a^
	Mean (SD)	85.3 (105.4)
	Median (IQR)	54 (38.5-94.5)
	Range	10-743
**Age (y)**		
	Mean age range	15.0-47.7
	Range across all studies	12-85
**Population in studies, n (%)**		
	Nonclinical (general population)	14 (26)
	University students	12 (23)
	Clinical (inpatient)	10 (19)
	Clinical (outpatient)	8 (15)
	Mixed (clinical: inpatient and outpatient)	1 (2)
	Mixed (clinical and nonclinical)	8 (15)
**Sampling protocol in studies, n (%)**		
	Daily diary (mixture of random and specific times)	13 (25)
	Signal contingent (pseudorandom)	33 (62)
	Signal contingent (specific times)	4 (8)
	Mixed (signal and event contingent)	2 (4)
	Event contingent	1 (2)
**Number of assessments per day** **in studies, n (%)**		
	1 (daily diary study or aggregated measure used)	15 (28)
	2-4	16 (30)
	5-10	22 (42)
**Study duration (d)**		
	Mean (SD)	19.9 (18.6)
	Median (range)	14.0 (3.4-90)
**Method of assessment** **in studies, n (%)**		
	Smartphone app	30 (57)
	Web link to surveys sent via SMS text message	10 (19)
	Web link to surveys sent via email	4 (8)
	PDA	2 (4)
	Other (eg, preprogrammed smartphone or iPad)	1 (2)
	Smartwatch	1 (2)
	Phone call (telephone interview)	1 (2)
	Paper (prompted by pager)	1 (2)
	Not reported	3 (6)

^a^69.8% female (women, female gender identity, or assigned female at birth where gender identity was not reported).

The sample sizes ranged from 10 to 743 participants (mean sample size 85.3, SD 105.4; median 54, IQR-38.5-94.5). Most studies (47/53, 89%) included largely female samples (69.8% of the total combined sample were female); in 4% (2/53) of the studies, all participants were female or women [22,23]; in 8% (4/53) of the studies, participants were mostly male or men [24-27]; 28% (15/53) of the studies included a small number of transgender, nonbinary, or gender nonconforming participants; and, in 2% (1/53) of the studies, all participants were transgender or gender diverse [28]. In 55% (29/53) of the studies, most participants were White; 19% (10/53) of the studies did not report the participants’ race or ethnicity. The mean sample age ranged from 15.0 to 47.7 years, with 28% (15/53) of the studies using a sample of young people (aged ≤25 years). In 26% (14/53) of the studies, participants were recruited from the general population, with 23% (12/53) of the studies recruiting from universities and the samples in the remaining studies being recruited from clinical settings or a mix of clinical and community settings.

Approximately a quarter of the studies (15/53, 28%) adopted a daily diary design with 1 assessment per day. Of the remaining 72% (38/53) of the EMA studies, the number of measurements ranged from 2 to 10 per day (mean 4.2, SD 2.3 measurements), and most (33/53, 62%) were delivered using a signal-contingent sampling scheme at pseudorandomized intervals. Study duration ranged from 3.4 to 90 days (mean 19.8, SD 18.5; median 14.0 days), with some studies (6/53, 11%) reporting varying durations based on length of hospitalization [24,25,29-32]. In the 85% (45/53) of the studies in which study duration was consistent for all participants, we found a small negative correlation (*r*=–0.14) between study duration (in days) and the number of assessments per day. EMA smartphone apps were most often used for data collection, including Illumivu, MetricWire, and movisensXS.

### Which of the Key Constructs in the IMV Model Were Assessed in EMA Studies, and How Were They Assessed?

In this section, we refer to the 58 individual papers. In total, 3% (2/58) of the papers [25,31] each reported 2 independent samples. Several other papers (11/58, 19%) reported the same sample but different IMV constructs [33,34]. A total of 45% (26/58) of the papers reported more than one IMV construct. In 12% (7/58) of the papers [26,31,35-39], the study used a signal-contingent (pseudorandom) sampling scheme with multiple daily assessments, but one or more IMV constructs were assessed once per day, or an aggregated daily measure was used.

Across the 58 papers included in this review, the motivational moderators in the IMV model were most frequently assessed in EMA studies (Table 2). The constructs measured most frequently were thwarted belongingness (24/58, 41% of the papers), positive or negative thoughts about the future (20/58, 34% of the papers), and perceived burdensomeness (16/58, 28% of the papers). The least frequently measured constructs were humiliation, social problem-solving, mental imagery, physical pain sensitivity, and fearlessness about death. None of the included papers measured memory biases, goals, norms, or resilience using EMA.

**Table 2:** Integrated motivational-volitional model constructs measured using intensive longitudinal methods by the number of assessments per day^a^.

			Papers (n=58), n (%)						
			1 assessment per day (daily diary)		2-4 assessments per day		5-10 assessments per day		Total
**Motivational phase**									
	Defeat	0 (0)		0 (0)		3 (5)		3 (5)	
	Humiliation	0 (0)		1 (2)		2 (4)		3 (5)	
	Entrapment	1 (2)		1 (2)		3 (5)		5 (9)	
**Threat-to-self moderators**									
	Coping	4 (7)		1 (2)		2 (4)		7 (12)	
	Rumination	2 (4)		2 (4)		4 (7)		8 (14)	
	Social problem-solving	1 (2)		0 (0)		1 (2)		2 (4)	
**Motivational moderators**									
	Future thoughts	5 (9)		6 (10)		9 (16)		20 (34)	
	Perceived burdensomeness	6 (10)		6 (10)		4 (7)		16 (28)	
	Thwarted belongingness	8 (14)		9 (16)		7 (12)		24 (41)	
	Social support	3 (5)		0 (0)		2 (4)		5 (9)	
**Volitional moderators**									
	Impulsivity	3 (5)		2 (4)		2 (4)		8 (14)^b^	
	Mental imagery	1 (2)		2 (4)		0 (0)		3 (5)	
	Physical pain sensitivity	3 (5)		1 (2)		1 (2)		6 (10)^b^	
	Fearlessness about death	2 (4)		1 (2)		1 (2)		4 (7)	
	Access to means	0 (0)		1 (2)		1 (2)		2 (4)	

^a^Suicidal thoughts and behaviors are not included in our results as these outcomes have been widely reported and discussed in other reviews of ecological momentary assessment studies.

^b^Total includes 1 event contingent study.

### Do Different Constructs From the IMV Model Show Fluctuation in Daily Life, and What Is Their Within-Person Variability?

#### Overview

ICCs were available in 78% (45/58) of the papers. These estimates varied substantially but, for most constructs, showed an overall pattern of at least moderate within-person variance (Table 3). A small number of constructs showed levels of within-person variance of <20% across a small number of papers (6/58, 10%), suggesting a more stable and traitlike construct in these particular samples.

There was variability across studies measuring the same IMV construct; however, no consistent patterns were observed in comparisons between IMV constructs measured in clinical versus community populations or in comparisons between different sampling frequency (number of assessments per day; see Multimedia Appendix 2 [22-79] for full details). The findings are described for each construct in the following sections and summarized in Tables 3 and 4.

**Table 3:** Proportion of within-person variance reported for each integrated motivational-volitional model construct by sample type^a^.

		Range across all studies (%)^b^	
		Clinical sample	Community sample
**Motivational phase**			
	Defeat	53 (n=1)	48 (n=1)
	Shame (humiliation)	18-89 (n=3)	—^c^
	Entrapment	39-48 (n=2)	46-63 (n=2)
**Threat-to-self moderators**			
	Coping	49-96 (n=3)	28-42 (n=1)
	Rumination	41-84 (n=5)	20-78 (n=4)
	Social problem-solving	34-75 (n=2)	—
**Motivational moderators**			
	Future thoughts	26-56 (n=11)	22-70 (n=6)
	Perceived burdensomeness	14-60 (n=11)	37-47 (n=2)
	(Thwarted) belongingness	4-57 (n=15)	33-90 (n=9)
	Social support	19-98 (n=5)	22-56 (n=3)
**Volitional moderators**			
	Impulsivity	25-78 (n=4)	59 (n=1)
	Mental imagery	64 (n=1)	56-79 (n=2)
	Physical pain sensitivity	48-74 (n=3)	29-61 (n=2)
	Fearlessness about death	53 (n=1)	12-31 (n=2)
	Access to means	—	34-45 (n=2)

^a^Within-person variance=1 – intraclass correlation coefficient.

^b^Number of studies indicated in parentheses.

^c^No studies reporting within-person variance.

**Table 4:** Overview of the included studies.

Study	Report type	Country	Sample type	Sample size, N	Age (y), mean (SD)	Gender or sex	Ethnicity or race	Mental health profile	Study duration (days)	Assessments per day, N	Compliance (%), mean (SD)	Constructs measured	ICC^a^ reported
Aadahl et al [48], 2021	Peer-reviewed article	United Kingdom	Mixed (clinical and community)	27	34.2 (13.9)	66% female; 34% male	93% White British; 7% White other	Recent SI^b^; 26% personality disorder; 45% affective disorder; 7% psychotic disorder; 3% eating disorder; 19% not stated	7	6	49	Defeat, entrapment, and hopelessness	No ICCs reported
Al-Dajani and Czyz [41], 2022	Peer-reviewed article	United States	Clinical (inpatient)	78	15.2 (1.4)	68% assigned female at birth	83% White; 6% Black; 5% Asian; 5% American Indian or Alaska Native; 4% other	Recent SI or SA^c^	28	1	72	Perceived burdensomeness, peer belongingness, and family belongingness	Perceived burdensomeness: 0.40; peer belongingness: 0.59; family belongingness: 0.43
Al-Dajani and Uliaszek [49], 2021	Peer-reviewed article	United States	Mixed clinical and nonclinical (including university students)	39	30.9 (8.8)	67% female; 26% male; 5% nonbinary or transgender	49% White; 19% Black; 16% other; 10% East Asian; 8% South Asian	59% lifetime SA	14	4	68	Hopelessness	0.49
Al-Dajani et al [43], 2022 (Same sample as the study by Al-Dajani and Czyz [41], 2022	Peer-reviewed article	United States	Clinical (inpatient)	78	15.2 (1.4)	68% assigned female at birth	83% White; 6% Black; 5% Asian; 5% American Indian or Alaska Native; 4% other	Recent SI or SA	28	1	72	Coping (personal support, professional support, noncognitive, cognitive, perceived helpfulness, and total strategies used)	Coping—personal support: 0.49; coping—professional support: 0.27; coping—noncognitive: 0.38; coping—cognitive: 0.55; coping—perceived helpfulness: 0.42; coping—total strategies used: 0.51
Ammerman et al [50], 2017	Peer-reviewed article	United States	Nonclinical	51	28.8 (9.8)	75% female	52% African American; 33% White; 10% Asian; 6% other	65% lifetime NSSI^d^; 100% BPD^e^ and depressive disorder	7	4	Not reported	Impulsivity	No ICCs reported
Baryshnikov et al [29], 2024	Peer-reviewed article	Finland	Clinical (inpatient)	67	37.3 (12.5)	66% female; 27% male; 7% other	Not reported	37% suicidal behavior; 100% unipolar depression	Varied (mean 3.4 days)	3	Not reported	Hopelessness	No ICCs reported
Bayliss et al [34], 2024	Peer-reviewed article	Australia	Nonclinical	75	36.5 (10.8)	64% female; 24% male; 12% other	Not reported	67% lifetime SA	14	4	74.5 (0.2)	Mental imagery, fearlessness about death, pain sensitivity, and access to means	Mental imagery: 0.28; fearlessness about death: 0.69; pain sensitivity: 0.71; access to means: 0.55
Bentley et al [24], 2021	Peer-reviewed article	United States	Clinical (inpatient)	83	38.4 (13.6)	52% male; 42% female; 4% transgender; 2% other	83% European descent; 5% Black or African American; 5% Asian; 6% other	100% recent SI or SA	Varied (mean 8.52, SD 5.73; range: 2-46 days)	4	52	Humiliation (shame)	0.82
Ben-Zeev et al [51], 2012	Peer-reviewed article	United States	Clinical (inpatient)	31	39.3 (11.0)	77% female	67% White; 13% African American; 3% Latinx; 17% other	58% lifetime SA; 100% depressive disorder	7	6	Not reported	Helplessness, hopelessness	No ICCs reported
Burke et al [52], 2021	Peer-reviewed article	United States	University students	60	20.1 (2.1)	92% female	68% White; 20% Asian; 7% mixed; 3% other	100% lifetime history of repetitive NSSI	10	3	89	Impulsivity	No ICCs reported
Christensen et al [53], 2023	Peer-reviewed article	United States	Nonclinical	93	23.5 (4.3)	14% cisgender men; 56% cisgender women; 5% transgender men; 23% gender queer or gender nonconforming; 2% another gender identity	67% non-Hispanic or Latinx White; 14% Hispanic or Latinx White; 3% Black; 6% Asian; 10% multiracial	100% recent NSSI urges	7-14	6	76	Social support	0.78
Cloos et al [22], 2020	Peer-reviewed article	Germany	University students	19	24.6 (4.5)	100% female	Not reported	100% recent NSSI; 89% personality disorder; 95% affective disorder	10	1	99	Entrapment, mental imagery compellingness, mental imagery vividness, mental imagery controllability, mental imagery nowness, mental imagery distress, and mental imagery comfort	Entrapment: 0.37; mental imagery compellingness: 0.21; mental imagery vividness: 0.29; mental imagery controllability: 0.22; mental imagery nowness: 0.29; mental imagery distress: 0.33; mental imagery comfort: 0.44
Coppersmith et al [54], 2019	Peer-reviewed article	United States	Nonclinical	53	23.5 (4.3)	77% female	75% White; 8% Asian; 2% Black or African American; 15% other	100% past-year SA	28	1	67% completed at least 14 days of responses	Social support	0.44
Czyz et al [44], 2019	Peer-reviewed article	United States	Clinical (inpatient)	34	15.5 (1.4)	77% female	85% White; 9% Black or African American;9% Asian	100% recent SI or SA; 85% depressive disorder; 71% anxiety disorder; 18% ADHD^f^	28	1	69	Hopelessness, perceived burdensomeness, and connectedness	Hopelessness: 0.67; perceived burdensomeness: 0.69; connectedness: 0.63
Czyz et al [45], 2019 (Same sample as the study by Czyz et al [44], 2019)	Peer-reviewed article	United States	Clinical (inpatient)	34	15.5 (1.4)	77% female	85% White; 9% Black or African American;9% Asian	100% recent SI or SA; 85% depressive disorder; 71% anxiety disorder; 18% ADHD	28	1	69	Coping (number of strategies used)	No ICCs reported
Czyz et al [42], 2021 (Same sample as the study by Al-Dajani and Czyz [41], 2022	Peer-reviewed article	United States	Clinical (inpatient)	78	15.2 (1.4)	68% assigned female at birth	83% White; 6% Black; 5% Asian; 5% American Indian or Alaska Native; 4% other	Recent SI or SA	28	1	72	Hopelessness, perceived burdensomeness, connectedness to friends, connectedness to family, and rumination	Hopelessness: 0.58; perceived burdensomeness: 0.62; connectedness to friends: 0.44; connectedness to family: 0.59; rumination: 0.47
Czyz et al [55], 2023	Peer-reviewed article	United States	Clinical (outpatient)	102	20.9 (2.1)	81.4% female; 18.6% male	75% White; 9% more than one category; 6% Asian; 5% Black or African American; 5% other	100% recent SI or SA	56	4	64	Rumination, hopelessness, perceived burdensomeness, thwarted belongingness (closeness to others), and coping	Rumination: 0.59; hopelessness: 0.73; perceived burdensomeness: 0.71; thwarted belongingness (closeness to others): 0.59; coping: 0.48\
Defayette et al [56], 2023	Peer-reviewed article	United States	University students	42	19.6 (1.3)	Sex at birth: 83.3% female and 16.7% male; gender identity: 73.8% women, 16.7% men, and 9.5% nonbinary	45% White; 17% African American; 17% Asian; 14% multiracial; 7% other	100% recent SI	28	6	72 (29.6)	Thwarted belongingness (social exclusion)	No ICCs reported
Ewing and Hamza [57], 2024	Peer-reviewed article	Canada	University students	160	19.7 (1.8)	83% female; 12% male; 5% transgender, unsure, nonbinary, or agender persons	44% White; 22% East Asian; 11% South Asian; 23% Filipino, Latin American, Black, Arab or West Asian, South East Asian, or Aboriginal	100% recent NSSI urges and past-year NSSI	14	1	89	Coping (problem focused, avoidant, emotion focused, and socially supported)	Coping—problem focused: 0.58; coping—avoidant: 0.63; coping—emotion focused: 0.72; coping—socially supported: 0.63
Gerner et al [58], 2023	Peer-reviewed article	United States	University students	43	19.1 (1.3)	70% women; 14% men; 12% gender nonconforming; 5% not listed	63% White; 21% Black or African American; 7% Asian or Asian American; 5% Latinx; 15% biracial	100% recent SI	10	5	86	Thwarted belongingness, perceived burdensomeness, and hopelessness	Thwarted belongingness: 0.64; perceived burdensomeness: 0.53; hopelessness: 0.37
Glenn et al [59], 2022	Peer-reviewed article	United States	Clinical (outpatient)	48	15.0 (1.6)	65% female; 17% male; 19% nonbinary	77% White; 14% Hispanic; 10% mixed; 8% Black; 2% American Indian	100% lifetime SI; 85% lifetime SA; 94% anxiety disorder; 28% ADHD; 83% major depressive disorder	28	3	Not reported	Thwarted belongingness	8 items ranging from 0.67 to 0.78
Hallard et al [60], 2021	Peer-reviewed article	United Kingdom	Clinical (inpatient) and nonclinical	24	35.3 (14.3)	67% female	92% White British; 8% White other	100% recent SI; 79% lifetime SA; 50% mood disorder; 30% personality disorder	6	7	48	Rumination	No ICCs reported
Hallensleben et al [46], 2019	Peer-reviewed article	Germany	Clinical (inpatient)	79	37.6 (14.3)	72% female	Not reported	100% lifetime SI; 34% lifetime SA; 87% depressive disorder	6	10	90	Hopelessness, perceived burdensomeness, and thwarted belongingness	Hopelessness: 0.74; perceived burdensomeness: 0.66; thwarted belongingness: 0.57
Harper [61], 2019	Thesis	United States	University students	145	20.1 (5.4)	72% female; 27% male	46% White; 33% African American; 15% Hispanic; 8% Asian	Not reported	7	3	85	Thwarted belongingness (loneliness)	0.50
Hughes et al [62], 2019	Peer-reviewed article	United States	Nonclinical	47	19.1 (1.8)	62% female; 30% male; 2% transgender	38% White; 19% Asian; 17% Hispanic; 15% Black or African American; 11% mixed	100% recent SH^g^	14	5	84% of participants had at least 80% compliance	Rumination	0.70
Jacobucci et al [63], 2023	Peer-reviewed article	United States	Nonclinical	35	25.9 (5.8)	63% identified as female; 20% identified as male; 14% identified as transgender and other	69% identified as White; 6% identified as Black; 11% Identified as Asian; 12% identified as other or more than one race	100% past-year SHTBs; 70% “seeing someone for emotional, psychiatric or substance use problems”	30	4	61	Perceived burdensomeness, and thwarted belongingness	No ICCs reported
Jeong et al [27], 2021	Peer-reviewed article	South Korea	Nonclinical	23	40.0 (8.7)	78% male	Not reported	Not reported	15	1	86	Impulsivity	0.41
Kaurin et al [64], 2022	Peer-reviewed article	United States	Clinical and nonclinical	186	33.7 (9.4)	80% female	76% White; 15% Black or African American; 4% Asian; 3% Pacific Islander; 2% other	56% lifetime SA; 82% BPD	21	Event contingent	Not reported	Impulsivity (during a social interaction)	0.54
Kaurin et al [47], 2023	Peer-reviewed article	United States	Clinical and nonclinical	153	33.6 (9.6)	81% female	Not reported	69% lifetime SA; 100% BPD	21	6	78	Impulsivity	No ICCs reported
Kellerman et al [30], 2022	Peer-reviewed article	United States	Clinical (inpatient)	118	15.8 (1.8)	80% female	81%non-Hispanic White; 4% Asian; 4% African American; 4% Hispanic	87% lifetime SI; 63% lifetime NSSI; 54% lifetime SA; 77% depressive disorder; 49% anxiety disorder	Varied (mean 6.1, SD 6.1 days)	1	Not reported	Social support from staff, social support from other patients, social support from family members, and social support from friends	Social support from staff: 0.71; social support from other patients: 0.73; social support from family members: 0.74; social support from friends: 0.81
Kirtley et al [38], 2022	Peer-reviewed article	Belgium	Nonclinical	743	16.9 (2.4)	59% female	Not reported	7% recent SI	6	10	70	Short-term future thinking (once per day)	0.30
Kleiman et al [25], 2017	Peer-reviewed article	Global	Nonclinical	54	23.2 (5.3)	80% female	72% European descent; 7% Hispanic; 7% Asian; 14% other	100% past-year SA	28	4	63	Hopelessness, loneliness, and perceived burdensomeness	Hopelessness: 0.57; loneliness: 0.49; perceived burdensomeness: 0.58
Kleiman et al [25], 2017	Peer-reviewed article	United States	Clinical (inpatient)	36	47.7 (13.1)	44% female	82% European descent; 6% Hispanic; 6% Asian; 6% other	100% recent SA or SI	Varied (mean 10.3, SD 6.5 days)	4	62	Hopelessness, and thwarted belongingness (loneliness)	Hopelessness: 0.66; thwarted belongingness (loneliness): 0.61
Krall et al [31], 2024	Peer-reviewed article	United States	University students	129	20.0 (1.6)	76% female biological sex assigned at birth; 24% male biological sex assigned at birth	49 % White; 38% Asian; 7% Black or African American; all others endorsed another or multiple races	100% SI	56	7	64	Pain (once per day) and hopelessness	Pain : 0.39; hopelessness: 0.78
Kudinova et al [65], 2023	Peer-reviewed article	United States	Clinical (inpatient)	158	15.2 (1.4)	68% were assigned female sex at birth; 61% identified as female; 32% identified as male	1% Asian; 9% Black or African American; 1% American Indian or Alaska Native; 66% White; 15% identified with more than one race	100% recent SI; 86% depressive disorder; 59% anxiety disorder	21	5	45% provided at least one response per day	Humiliation (shame)	0.11
Kuehn [66], 2022	Thesis	United States	Clinical (outpatient) and nonclinical	60	18.6 (1.3)	77% female sex; 23% male sex	53% self-identified as White; 12% self-identified as Hispanic or Latinx; 10% reported a mixed ethnicity; 20% self-identified as Asian; 3% self-identified as Black or African American; 2% self-identified as Middle Eastern	100% recent SH, SI or past-year SA	14	5	87	Coping (cognitive reappraisal, self-invalidation, suppression, distraction, acceptance, and avoidance), rumination, problem-solving, social support, impulsivity, and shame	Coping—cognitive reappraisal: 0.31; coping—self-invalidation: 0.32; coping—suppression: 0.31; coping—distraction: 0.24; coping—acceptance: 0.25; coping—avoidance: 0.04; rumination: 0.16; problem-solving: 0.25; social support: 0.02; impulsivity: 0.75; shame: 0.38
López et al [67], 2023	Peer-reviewed article	United States	University students	49	19.7 (1.6)	71% female; 29% male	45% White; 12% African American; 22% Asian; 8% multiracial; 12% other	100% recent SI	28	6	51	Thwarted belongingness	0.39
Lucht et al [35], 2022 (same sample as in the study by Hallensleben et al [46], 2019)	Peer-reviewed article	Germany	Clinical (inpatient)	79	37.6 (14.3)	72% female	Not reported	100% lifetime SI; 34% lifetime SA; 87% depressive disorder	6	10	90	Impulsivity (once per day)	4 items ranging from 0.22 to 0.36
MacNeil et al [68], 2023	Peer-reviewed article	Canada	Clinical (outpatient) and nonclinical	55	15.6 (1.6)	75% female	75% White	44% major depressive disorder	10	1	Not reported	Thwarted belongingness and perceived burdensomeness	Thwarted belongingness: 0.53; perceived burdensomeness: 0.67
Mitchell et al [69], 2023	Peer-reviewed article	United States	University students	41	19.3 (2.0)	17% male; 83% female	34% Asian; 32% White; 15% multiracial; 12% Black; 7% Hispanic or Latinx	29% lifetime NSSI; 32% lifetime SA	5	1	Not reported	Rumination	0.22
Molaie [70], 2022	Thesis	United States	University students	197	19.4 (1.8)	79% of participants identified as female; 19% identified as male; 0.5% identified as nonbinary or gender nonconforming	49% White; 23% Hispanic or Latinx; 14% Asian American; 7% multiethnic; 4% African American; 2% Native Hawaiian or Pacific Islander; 1% Middle Eastern; 2% other	Not reported	14	1	79	Thwarted belongingness	0.67
Mournet et al [39], 2022	Peer-reviewed article	United States	University students	74	19.4 (1.0)	70% cisgender female; 26% cisgender male; 3% transgender female; 1% nonbinary; 1% chose not to disclose	50% Asian; 31% White; 5% African American or Black; 1% American Indian or Alaska Native; the remainder endorsed multiple races or chose not to disclose	100% recent SI	56	6	70	Thwarted belongingness (loneliness; once per day), and perceived burdensomeness (once per day)	No ICCs reported
Nuij et al [26], 2022	Peer-reviewed article	The Netherlands	Clinical (outpatient)	17	32.1 (9.2)	47% female	Not reported	100% recent SI; 94% depressive disorder; 53% lifetime SA	90	4	18	Perceived burdensomeness, thwarted belongingness (once per day), entrapment, future thoughts (once per day), hopelessness, mental imagery, impulsivity (once per day), problem-solving and coping (once per day), and rumination	Perceived burdensomeness: 0.86; thwarted belongingness (once per day): —; entrapment: 0.52: future thoughts (once per day): 0.60; hopelessness: 0.44; mental imagery: 0.36; impulsivity (once per day): 0.75; problem-solving or coping (once per day): 0.66; rumination: 0.47
Parrish et al [71], 2021	Peer-reviewed article	United States	Clinical (outpatient)	96	43.9 (11.2)	55% female	48% Black or African American; 28% White; 24% Hispanic; 24% other	100% current diagnosis of schizophrenia, schizoaffective disorder, bipolar disorder with psychotic features, or major depressive disorder with psychotic features	10	3	81 (19.9)	Perceived burdensomeness and thwarted belongingness	Perceived burdensomeness: 0.69; thwarted belongingness: 0.59
Peters et al [32], 2022	Peer-reviewed article	Canada	Clinical (inpatient)	39	36.3 (13.0)	69% female	Not reported	100% recent SI	Varied (mean 12 days)	3	Not reported	Thwarted belongingness (social connectedness)	0.56
Reeves [72], 2022	Thesis	United States	Clinical (outpatient)	10	Not reported	50% gender nonconforming or variant; 40% cisgender female; 10% cisgender male	60% White or European descent; 10% Chinese; 10% Middle Eastern; 20% other	100% lifetime SI; 100% depressive disorder	14	9	82	Thwarted belongingness, perceived burdensomeness, and hopelessness	Thwarted belongingness: 0.74; perceived burdensomeness: 0.44; hopelessness: 0.48
Rogers [73], 2023	Peer-reviewed article	United States	Nonclinical	237	27.1 (8.6)	62% female; 16% nonbinary; 9% transgender male; 7% male; 2% transgender female	87% White or European American; 4% Black or African American; 7% Hispanic or Latino or a; 4% Asian; 3% other	100% recent SI; 68% lifetime SA	14	6	69	Rumination, suicide-specific rumination, thwarted belongingness, perceived burdensomeness, hopelessness, fearlessness about death, and access to means	Rumination: 0.56; suicide-specific rumination: 0.80; thwarted belongingness: 0.54; perceived burdensomeness: 0.63; hopelessness: 0.63; fearlessness about death: 0.88; access to means: 0.66
Selby et al [74], 2019	Peer-reviewed article	United States	Clinical (outpatient) and nonclinical	47	19.1 (1.8)	68% female; 30% male; 2% transgender	38% White; 15% African American; 19% Asian; 17% Hispanic or Latino; 11% mixed	100% recent NSSI	14	5	84% had compliance of >80%	Physical pain (event contingent)—pre-NSSI pain rating	0.26
Silva et al [75], 2022	Peer-reviewed article	United States	Clinical (outpatient)	16	43.8 (10.8)	81% female	100% Hispanic or Latina	69% major depressive episode; 25% PTSD^h^; 25% GAD^i^; 19% psychotic disorder	14	4	74 (17.6)	Thwarted belongingness, perceived burdensomeness, emotional loneliness, and social loneliness	Thwarted belongingness: 0.85; perceived burdensomeness: 0.81; emotional loneliness: 0.85; social loneliness: 0.96
Spangenberg et al [36], 2019 (same sample as in the study by Hallensleben et al [46], 2019)	Peer-reviewed article	Germany	Clinical (inpatient)	79	37.6 (14.3)	72% female	Not reported	100% lifetime SI; 34% lifetime SA; 87% depressive disorder	6	10	90	Fearlessness about death (once per day) and pain tolerance (once per day)	Fearlessness about death (once per day): 0.47; pain tolerance (once per day):0.52
Stanley et al [76], 2021	Peer-reviewed article	United States	Clinical (outpatient)	50	30.6 (11.0)	86% female	56% White	100% BPD; 100% current SI, recent NSSI, or recent SA	7	6	70	Coping strategies (engagement and effectiveness)	No ICCs reported
Stenzel et al [77], 2020	Peer-reviewed article	Germany	Nonclinical	61	24.2 (7.0)	89% women	Not reported	No current mental illness	7	5	82	Defeat and entrapment	Defeat: 0.52; entrapment: 0.54
Tsypes et al [37], 2022 (same sample as in the study by Kaurin et al [47], 2023)	Peer-reviewed article	United States	Clinical and nonclinical	153	33.6 (9.6)	81% female	Not reported	69% lifetime SA; 100% BPD	21	6	78	Reasons for living (once per day)	0.63
Turner et al [33], 2016	Peer-reviewed article	Canada	Nonclinical	60	23.4 (4.3)	85% female	53% White; 18% East Asian; 8% Southeast Asian; 3% Native Canadian; 2% Black or African Canadian; 2% Hispanic or Latina or Latino	100% recent, repeated NSSI	14	1	88	Perceived social support	0.56
Turner et al [40], 2019 (same sample as in the study by Turner et al [33], 2016)	Peer-reviewed article	Canada	Nonclinical	60	23.4 (4.3)	85% female	53% White; 18% East Asian; 8% Southeast Asian; 3% Native Canadian; 2% Black or African Canadian; 2% Hispanic, Latina, or Latino	100% recent, repeated NSSI	14	1	88	Daily coping strategies	No ICCs reported
van Ballegooijen et al [78], 2022	Peer-reviewed article	United Kingdom	Clinical and nonclinical	51	35.5 (12.8)	67% women	Not reported	71% lifetime SA	7	6	87	Defeat and entrapment	Defeat: 0.47; entrapment: 0.58
Victor et al [23], 2019	Peer-reviewed article	United States	Nonclinical	63	22.0 (1.6)	100% women	71% African American; 24% non-Hispanic White	100% lifetime SI	21	6	75	Thwarted belongingness (interpersonal stress) and thwarted belongingness (rejection)	Thwarted belongingness (interpersonal stress) : 0.10; Thwarted belongingness (rejection): 0.17
Wolford‐Clevenger et al [79], 2020	Peer-reviewed article	United States	University students	206	19.1 (2.4)	73% women	82% White; 8% Hispanic or Latino or Latina	100% lifetime SI; 25% lifetime SA	90	1	58 (after 30 days); 27 (after 90 days)	Thwarted belongingness, perceived burdensomeness, hopelessness, and capability for suicide (pain tolerance and fearlessness about death)	No ICCs reported
Wolford‐Clevenger et al [28], 2021	Peer-reviewed article	United States	Nonclinical (including university students)	38	28.6	100% transgender, gender diverse, of transgender experience, or having transitioned (37% female or transgender women)	84% non-Hispanic White	Not reported	30	1	73	Hopelessness and thwarted belongingness (social connectedness)	No ICCs reported

^a^ICC: intraclass correlation coefficient.

^b^SI: suicidal ideation.

^c^SA: suicide attempt.

^d^NSSI: nonsuicidal self-injury.

^e^BPD: borderline personality disorder.

^f^ADHD: attention-deficit/hyperactivity disorder.

^g^SH: self-harm.

^h^PTSD: posttraumatic stress disorder.

^i^GAD: generalized anxiety disorder.

#### Motivational Phase

##### Defeat

In total, 5% (3/53) of the studies [48,77,78] measured defeat, each with 5 to 10 assessments per day. ICCs were available in 67% (2/3) of these studies, with a 48% within-person variance reported across a community sample of 61 young adults, mostly women [77], and a 52% within-person variance reported across a mixed clinical and community sample of 51 adults [78]. Defeat was measured using a single item and operationalized variously as “powerless” [48] or “emotionally defeated” [78] (see Multimedia Appendix 3 [22-79] for details).

##### Humiliation

A total of 6% (3/53) of the studies [24,65,68] measured humiliation. Bentley et al [24] reported an 18% within-person variance across a clinical sample of 83 (mostly male) adults with 4 assessments per day, whereas Kudinova et al [65] reported an 89% within-person variance in “anger at self” across a clinical sample of 158 (mostly female) adolescents. In a mixed sample of mostly female young adults (N=60), Kuehn [66] reported a 62% within-person variance with 5 assessments per day. See Multimedia Appendix 3 [22-79] for the item wording.

##### Entrapment

In total, 9% (5/53) of the studies [22,26,48,77,78] measured entrapment using 4 to 6 assessments per day or once per day in a daily diary study [22]. While Cloos et al [22] reported a 63% within-person variance in a community sample of 19 young women, 6% (3/53) of the EMA studies reported similar proportions of within-person variance (42%-48%) across a small adult clinical sample [26], a community sample of 61 young (mostly female) adults [77], and a mixed clinical and community sample of 51 adults [78]. Entrapment was measured using 2 items in each study except for those by Nuij et al [26] and Cloos et al [22], who each used a single item (see Multimedia Appendix 3 [22-79] for the item wording).

#### Threat-to-Self Moderators

##### Coping

Using a range of sampling frequencies, 13% (7/53) of the studies measured coping [40,43,45,55,58,66,76], and we found substantial variation in how this construct was operationalized (Multimedia Appendix 3). A total of 8 coping strategies were grouped into personal support, professional support, and cognitive and noncognitive strategies in the study by Al-Dajani et al [43], with participants asked to report daily level of engagement with each group of coping strategies to deal with suicidal thoughts or stressful events (45%-73% within-person variance in a clinical, mostly female adolescent sample), overall perceived helpfulness (regardless of strategy; 58% within-person variance), and total coping strategies used (49% within-person variance). Czyz et al [55] used the same grouping of strategies assessed 4 times per day for 8 weeks but reported a single ICC for overall engagement (52% within-person variance in a young adult, mostly female clinical sample). Ewing and Hamza [57] provided separate ICCs for daily frequency of engagement in coping strategies grouped into problem-focused, avoidant, emotion-focused, and socially supported coping (28%-42% within-person variance in a predominantly female, young adult community sample). In a mixed, young adult sample, Kuehn [66] included rumination, problem-solving, and social support as coping strategies alongside cognitive reappraisal (69% within-person variance), self-invalidation (68% within-person variance), suppression (69% within-person variance), distraction (76% within-person variance), acceptance (75% within-person variance), and avoidance (96% within-person variance). In the study by Stanley et al [76], adult clinical participants were asked 6 times per day whether they had used each of the following strategies and rated their perceived effectiveness in reducing distress: keeping busy, socializing, positive thinking, doing something good for oneself, calming oneself, finding perspective, and sitting with one’s feelings until they passed. In a predominantly female community sample of 60 young adults, Turner et al [40] asked participants once per day whether they had used each of 15 strategies (grouped as problem focused, support seeking, and avoidant coping) to deal with a named problem or stressor encountered that day (reporting a 44% within-person variance).

##### Rumination

A total of 15% (8/53) of the studies measured rumination using both a daily diary design [41,69] and ≥4 assessments per day [26,55,57,60,66,73] using different operationalizations (Multimedia Appendix 3). The within-person variance differed substantially across studies. This was 53% when assessed once daily in a clinical adolescent sample [42] and when assessed 4 times per day in a clinical adult sample [26]. The within-person variance was 41% when assessed 4 times per day over 8 weeks in a clinical young adult, predominantly female sample [55]. Hughes et al [62] reported a lower within-person variance (30%) in a community sample of 47 young adults, whereas Kuehn [66] reported a much higher within-person variance (84%) in a mixed young adult sample measuring rumination as a binary coping strategy. In another sample of young adults in the community, Mitchell et al [69] reported similarly high within-person variance (78%). Rogers [73] measured both rumination and suicide-specific rumination in an adult community sample and found lower within-person variance in the latter (20% compared to 44%).

##### Social Problem-Solving

A total of 4% (2/53) of the studies measured social problem-solving; in both cases, this was operationalized as a form of coping. Kuehn [66] reported a 75% within-person variance in a mixed young adult (predominantly female) sample (N=60) measuring social problem-solving 5 times per day as a binary coping strategy. However, Nuij et al [26] reported a 44% within-person variance in a clinical adult sample assessing the construct once daily.

#### Motivational Moderators

##### Thoughts About the Future

Future thinking was measured in 38% (20/53) of the studies, most frequently operationalized as “hopelessness” and measured once per day [28,42,44,79] or 3 to 10 times per day [25,26,29,31,46,48,49,51,55,58,72,73]. The proportion of within-person variance reported differed substantially across studies measuring “hopelessness,” from 22% when assessed 7 times per day in a young adult community sample (N=129) [31] to 63% when assessed 5 times per day in a similar young adult community sample (N=43) [58].

Other studies operationalized the construct as short-term future thinking (70% within-person variance in a large adolescent community sample measured once each morning [38]), future thinking (40% within-person variance in a clinical adult sample measured once per day [26]), and a 6-item reasons-for-living scale (37% within-person variance in a clinical adult sample measured once per day [37]).

##### Perceived Burdensomeness

In total, 30% (16/53) of the studies measured perceived burdensomeness in daily diary studies [39,41,42,44,68,79] or using ≥3 assessments per day [25,26,46,54,58,63,71-73,75]. Operationalization of perceived burdensomeness was more homogeneous across studies than that of some of the other constructs (Multimedia Appendix 3).

The reported within-person variance ranged from 14% in a small clinical adult sample assessed 4 times per day (47% female) [26] to 60% in a clinical adolescent sample assessed 6 times per day (68% female [41]).

##### Thwarted Belongingness

Thwarted belongingness was the most frequently included IMV model construct across studies, with 45% (24/53) of the studies measuring thwarted belongingness in a daily diary design [28,39,41,44,48,55,68,70] or using ≥3 assessments per day [23,25,32,46,55,56,58,59,61,63,67,71-73,75]. The construct was variously referred to as “connectedness,” “closeness to others,” “social exclusion,” “loneliness,” and “thwarted belongingness.”

In total, 8% (2/24) of these studies [41,59] distinguished between peer and family belongingness. Gerner et al [58] reported a 36% within-person variance in a community sample of young adults with recent SI. Harper [61] measured loneliness in a community sample of young adults reporting a 50% within-person variance. López et al [67] measured “social rejection severity” in a community sample of young adults and reported a 41% within-person variance.

There was large variation in reported proportions of within-person variance. This ranged from 15% when measured as “emotional loneliness” (and just 4% when measured as “social loneliness”) 4 times per day in a small, Hispanic or Latino adult, predominantly female clinical sample [75]—suggesting more stable, traitlike constructs—to 83% and 90% when measured as “rejection” and “interpersonal stress” 6 times per day in a community sample of predominantly African American young women [23], suggesting more unstable, statelike constructs.

##### Social Support

Social support was operationalized differently across 9% (5/53) of the studies [30,33,53,54,66]. In the study by Kellerman et al [30], participants were asked to report how supported they felt by staff in the psychiatric unit in which they were hospitalized (29% within-person variance), other patients on the unit (27%), family members (32%), and friends outside the unit (19%). Meanwhile, Kuehn [66] included “social support” as a coping strategy, with participants asked whether they had or had not used this (98% within-person variance). Coppersmith et al [54] reported a 56% within-person variance in a sample of young adults reporting a past-year suicide attempt. Turner et al [33] reported a daily perceived support score averaging ratings from items regarding romantic partners, parents, and peers if the participant reported having contact with each since the previous assessment (44% within-person variance in a sample of young adults with NSSI thoughts or urges in the previous 2 weeks). Christensen et al [53] reported a 22% within-person variance in a community sample of young adults with past-month NSSI urges.

#### Volitional Moderators

##### Impulsivity

In total, 15% (8/53) of the studies assessed impulsivity once per day [26,27,35], ≥3 times per day [47,50,52,66], or using event-contingent sampling [64].

In a community sample of young adults, Kuehn [66] reported a 25% within-person variance using the following item: “When I am upset, I often act without thinking” (the wording was intended to assess momentary impulsivity, but a “global” measure was mistakenly implemented).

In a small, mostly male community sample of adults in South Korea with chronic pain, Jeong et al [27] reported a 59% within-person variance in impulsivity measured once per day. Lucht et al [35] measured impulsivity once per day in an adult sample of psychiatric inpatients, reporting a 64% to 78% within-person variance for each item. Nuij et al [26] measured impulsivity once per day, reporting a 25% within-person variance.

Kaurin et al [64] used an event-contingent sampling scheme to assess impulsivity during a social interaction using a single item. A 46% within-person variance was reported in a mostly female sample of participants drawn from a mixture of clinical and community sources, most of whom had a diagnosis of borderline personality disorder.

##### Mental Imagery

Mental imagery was measured in 6% (3/53) of the studies. Nuij et al [26] reported a 64% within-person variance in a small clinical sample, whereas Bayliss et al [34] reported a 72% within-person variance in an adult community sample.

Cloos et al [22] measured different aspects of mental imagery once daily in a small sample of young women with NSSI, most of whom were also diagnosed with a personality disorder. The within-person variance ranged from 56% (comfort) to 79% (compellingness).

##### Physical Pain Sensitivity

A total of 11% (6/53) of the studies (reported in 5/58, 9% of the papers) measured perceptions of pain or pain tolerance daily [31,36,79], 4 times per day [34], 7 times per day [31], or using an event-contingent design [74].

Krall et al [31] reported 4% (2/53) of the studies. The first reported a 61% within-person variance in physical pain over the course of the day in a community sample of young adults. The second reported a 76% within-person variance when measured 7 times per day in a largely female sample of outpatient adults with borderline personality disorder.

In a mixed sample of young people who reported NSSI in the previous 2 weeks, Selby et al [74] used an event-contingent design. If participants reported having engaged in NSSI at any assessment (up to 5 times per day), they were asked to rate their experience of physical pain before the NSSI episode. For pre-NSSI pain, a 74% within-person variance was reported.

Bayliss et al [34] reported a 29% within-person variance in a community sample, conceptualizing pain tolerance as “dispositional capability for suicide.”

Perceived pain tolerance was measured once daily by Spangenberg et al [36], with a 48% within-person variance reported in an adult sample of psychiatric inpatients with a current unipolar depressive disorder. Wolford-Clevenger et al [79] measured daily perceived pain tolerance in a (predominantly White and predominantly female) community sample of young adults but combined this with an item measuring daily fearlessness about death to produce a measure of “capability for suicide” (no ICC available).

##### Fearlessness About Death

A total of 8% (4/53) of the studies measured fearlessness about death [34,36,73] (see also the aforementioned study by Wolford-Clevenger et al [79]). Fearlessness about death was measured once daily by Spangenberg et al [36], with a 53% within-person variance reported in an adult sample of psychiatric inpatients with a current unipolar depressive disorder. Bayliss et al [34] reported a 31% within-person variance in an adult community sample, conceptualizing fearlessness about death as “acquired capability for suicide.”

Rogers [73] reported a 12% within-person variance when assessed 6 times per day in a community sample of adults with current SI.

##### Access to Means

Only 4% (2/53) of the studies [34,73] measured access to means, both in adult community samples, reporting a 34% within-person variance when operationalized as “physical distance to methods” [73] and a 45% within-person variance when operationalized as “practical capability for suicide” [34].

### What Is the Relationship Between the Different IMV Constructs and Suicidal and Nonsuicidal Thoughts and Behaviors in Daily Life?

#### Overview

The studies reported in 39 papers tested associations between IMV constructs and SHTBs in daily life; 14% (8/58) papers reported concurrent associations only, 28% (16/58) papers reported lagged associations only, and 26% (15/58) papers reported both concurrent and lagged associations. Where lagged associations were reported, lags varied from a few hours to a day. The studies varied regarding which SHTBs were tested, including SI, suicidal urge intensity, NSSI, NSSI urge, and suicidal behaviors. Table 5 summarizes papers reporting associations between IMV constructs and suicidal thoughts and behaviors in daily life, and Table 6 presents associations between IMV constructs and nonsuicidal SHTBs in daily life.

**Table 5:** Summary of papers reporting associations between integrated motivational-volitional (IMV) model constructs and suicidal thoughts and behaviors in daily life.

Study and IMV construct measured			Concurrent associations		Lagged associations		SHTBs^a^ measured	
**Aadahl et al [48], 2021**								SI^b^
	Defeat	β^c^=.21 (95% CI .11-.31); *P*<.001		—^d^				
	Hopelessness (future thoughts)	β=.18 (95% CI .08-.27); *P*<.001		—				
**Al-Dajani and Czyz [41], 2022**								Suicidal urge intensity
	Perceived burdensomeness	b^e^=0.45; SE 0.05; *P*<.001		b=0.14; SE 0.05; *P*=.008				
	Peer belongingness	b=–0.12; SE 0.03; *P*<.001		b=–0.04; SE 0.03; *P*=.14				
	Family belongingness	b=–0.14; SE 0.03; *P*<.001		b=0.02; SE 0.04; *P*=.68				
**Al-Dajani et al [43], 2022**								Suicidal urge intensity
	Coping	—		b=–0.02; SE 0.02; *P*=.24				
**Baryshnikov et al [29], 2024**								Suicidality
	Hopelessness (future thoughts)	—		β=.71 (95% CI .62-0.81)				
**Bayliss et al [34], 2024**								SI
	Mental imagery	OR^f^ 5.15 (95% CI 4.04-6.57); *P*<.001		OR 1.38 (95% CI 1.08-1.77); *P*<.01				
	Fearlessness about death	OR 1.29 (95% CI 0.95-1.75); *P*=.11		OR 1.22 (95% CI 0.89-1.68); *P*=.22				
	Pain sensitivity	OR 0.82 (95% CI 0.61-1.10); *P*=.19		OR 0.93 (95% CI 0.68-1.26); *P*=.71				
	Access to means	OR 0.82 (95% CI 0.63-1.07); *P*=.14		OR 0.92 (95% CI 0.71-1.19); *P*=.42				
**Ben-Zeev et al [51], 2012**								SI
	Helplessness	—		b=0.033; *P*=.09				
	Hopelessness (future thoughts)	—		b=0.006; *P*=.64				
**Coppersmith et al [54], 2019**								SI
	Social support	b=–0.41 (95% CI –0.52 to –0.30); *P*<.001		b=0.00 (95% CI –0.04 to 0.05); *P*=.86				
**Czyz et al [44], 2019**								SI frequency, duration, and urge severity
	Hopelessness (future thoughts)	SI frequency: b=0.37, β=.26, and *P*<.001; SI duration: b=–0.37, β=.22, and *P*<.001; SI urge severity: b=0.46, β=.20, and *P*<.001		SI frequency: b=0.01, β=.01, and *P*=.87; SI duration: b=–0.01, β=–.01, and *P*=.91; SI urge severity: b=0.10, β=.04, and *P*=.47				
	Perceived burdensomeness	SI frequency: b=0.11, β=.15, and *P*=.003; SI duration: b=0.16, β=.19, and *P*<.001; SI urge severity: b=0.25, β=.21, and *P*<.001		SI frequency: b=0.06, β=.08, and *P*=.16; SI duration: b=0.10, β=.12, and *P*=.04; SI urge severity: b=0.13, β=.12, and *P*=.05				
	Connectedness	SI frequency: b=–0.16, β=–.19, and *P*<.001; SI duration: b=–0.18, β=–.18, and *P*<.001; SI urge severity: b=–0.27, β=–.20, and *P*<.001		SI frequency: b=–0.06, β=–.07, and *P*=.17; SI duration: b=–0.07, β=–.07, and *P*=.16; SI urge severity: b=–0.09, β=–.07, and *P*=.21				
**Defayette et al [56], 2023**								SI
	Thwarted belongingness—social exclusion	b=0.04 (95% CI –0.001 to 0.07); *P*=.06; *d*=0.58		b=–0.003 (95% CI –0.02 to 0.01); *P*=.66				
**Gerner et al [58], 2023**								SI
	Thwarted belongingness	b=0.15 (95% CI 0.11-0.19); *P*<.001		b=0.06 (95% CI 0.02-0.11); *P*<.01				
	Perceived burdensomeness	b=0.08 (95% CI 0.04-0.12); *P*<.001		b=0.03 (95% CI –0.01 to 0.07); *P*>.05				
	Hopelessness (future thoughts)	b=0.25 (95% CI 0.22-0.28); *P*<.001		b=0.14 (95% CI 0.09-0.20); *P*<.001				
**Glenn et al [59], 2022**								SI
	Thwarted belongingness	—		Thwarted belongingness—family: β=.26 (*P*<.05); thwarted belongingness—friends: β=.31 (*P*<.05)				
**Hallard et al [60], 2021**								SI
	Rumination	—		β=.20 (95% CI .12-.27); *P*<.001				
**Hallensleben et al [46], 2019**								SI
	Hopelessness (future thoughts)	b=0.41 (95% CI 0.35-0.47); *P*<.001		b=0.08 (95% CI 0.02-0.18); *P*=.007				
	Perceived burdensomeness	b=0.09 (95% CI 0.05-0.13); *P*<.001		b=0.09 (95% CI 0.03-0.15); *P*=.001				
	Thwarted belongingness	b=0.04 (95% CI 0.00-0.08); *P*=.01		b=0.00 (95% CI –0.03 to 0.04); *P*=.99				
**Jacobucci et al [63], 2023**								SI
	Perceived burdensomeness	—		β=.16 (95% CI .01-.32)				
	Thwarted belongingness	—		β=.18 (95% CI .02-.34)				
**Kaurin et al [64], 2022**								SI
	Impulsivity—during a social interaction	b=0.01 (95% CI –0.00 to 0.01); β=.068		—				
**Kaurin et al [47], 2023**								SI
	Impulsivity	b=0.03 (95% CI 0.01-0.05)		—				
**Kleiman et al [25], 2017**								SI
	Hopelessness (future thoughts)	b=0.70 (95% CI 0.62-0.78); SE 0.04; *P*<.001		Controlling for SI at previous timepoint: b=0.07 (95% CI –0.05 to 0.20); SE 0.07; *P*=.27				
	Loneliness (thwarted belongingness)	b=0.26 (95% CI 0.19-0.33); SE 0.04; *P*<.001		Controlling for SI at previous timepoint: b=–0.04 (95% CI –0.14 to 0.07); SE 0.05; *P*=.51				
	Perceived burdensomeness	b=0.33 (95% CI 0.25-0.40); SE 0.04; *P*<.001		Controlling for SI at previous timepoint: b=0.03 (95% CI –0.08 to 0.14); SE 0.06; *P*=.62				
	Hopelessness (future thoughts)	b=0.92 (95% CI 0.76-1.08); SE 0.08; *P*<.001		Controlling for SI at previous timepoint: b=–0.03 (95% CI –0.24 to 0.19); SE 0.11; *P*=.81				
	Loneliness (thwarted belongingness)	b=0.16 (95% CI 0.01-0.31); SE 0.08; *P*=.04		Controlling for SI at previous timepoint: b=–0.10 (95% CI –0.29 to 0.09); SE 0.10; *P*=.32				
**Kuehn [66], 2021**								SI
	Coping	OR 1.31 (95% CI 1.15-1.49)		OR 1.31 (95% CI 0.93-1.84)				
	Impulsivity	OR 0.98 (95% CI 0.80-1.19)		—				
	Humiliation	—		OR 1.19 (95% CI 1.05-1.32)				
**Lucht et al [35], 2022**								SI
	Impulsivity	β=.12 (95% CI –.10 to .35); *P*=.27		—				
**Mournet et al [39], 2022**								SI
	Loneliness (thwarted belongingness)	OR 1.355 (95% CI 1.22-1.50); *P*<.001		OR 1.248 (95% CI 1.12-1.39); *P*<.001				
	Perceived burdensomeness	OR 1.289 (95% CI 1.19-1.40); *P*<.001		OR 1.103 (95% CI 1.01-1.21); *P*=.03				
**Peters et al [32], 2022**								Suicidality
	Social connectedness (thwarted belongingness)	Correlation=0.09 (*P*>.05)		—				
**Rogers [73], 2023**								Suicidal behaviors
	Rumination	OR 3.53 (95% CI 0.82-15.30); *P*=.09		OR 2.38 (95% CI 0.17-32.44); *P*=.52				
	Suicide-specific rumination	OR 3.85 (95% CI 0.96-15.46); *P*=.06		OR 2.26 (95% CI 0.21-24.00); *P*=.50				
	Thwarted belongingness	OR 1.87 (95% CI 0.35-9.93); *P*=.46		OR 1.85 (95% CI 0.08-40.39); *P*=.70				
	Perceived burdensomeness	OR 3.28 (95% CI 0.76-14.09); *P*=.11		OR 2.01 (95% CI 0.15-26.46); *P*=.59				
	Hopelessness (future thoughts)	OR 3.99 (95% CI 1.00-15.89); *P*=.05		OR 2.31 (95% CI 0.24-22.58); *P*=.47				
	Fearlessness about death	OR 1.81 (95% CI 0.56-5.87); *P*=.32		OR 1.61 (95% CI 0.20-14.08); *P*=.64				
	Access to means—physical	OR 1.37 (95% CI 0.36-5.25); *P*=.64		OR 1.41 (95% CI 0.17-11.59); *P*=.75				
	Access to means—psychological	OR 2.73 (95% CI 1.04-7.14); *P*=.04		OR 2.12 (95% CI 0.38-11.72); *P*=.39				
**Stanley et al [76], 2021**								SI
	Coping strategies (engagement and effectiveness)	—		Distraction or positive activity-based coping factor: b=–0.08 and *P*<.001; mindfulness-oriented coping factor: b=–0.03 and *P*=.03				
**Tsypes et al [37], 2022**								SI
	Reasons for living (future thoughts)	β=–.13 (95% CI –.18 to –.08)		β=–.05 (95% CI –.09 to –.01)				
**van Ballegooijen et al [78], 2022**								SI
	Entrapment	Correlation=0.27 (*P*<.001)		Lag 1 (2-4 h): correlation=0.10 (*P*=.01); lag 2 (5-7 h): correlation=0.10 (*P*=.03); lag 3 (8-10 h): correlation=0.11 (*P*=.02); lag 4 (11-13 h): correlation=0.12 (*P*=.04)				
	Defeat	Correlation=0.15 (*P*<.001)		Lag 1 (2-4 h): correlation=0.02 (*P*=.64); lag 2 (5-7 h): correlation=–0.02 (*P*=.62); lag 3 (8-10 h): correlation=0.02 (*P*=.70); lag 4 (11-13 h): correlation=0.04 (*P*=.50)				
**Victor et al [23], 2019**								Suicidal urges
	Criticism (thwarted belongingness)	—		β=.10 (95% CI –.01 to .21)				
	Rejection (thwarted belongingness)	—		β=.07 (95% CI –.05 to .18)				
**Wolford** **-** **Clevenger et al [79], 2020**								SI and suicidal behavior
	Thwarted belongingness	—		Passive SI: β=.03, SE 0.004, *t*=6.91, and *P*<.001; active SI: β=.003, SE 0.002, *t*=1.95, and *P*=.05				
	Perceived burdensomeness	—		Passive SI: β=.04, SE 0.005, *t*=7.69, and *P*<.001; active SI: β=.005, SE 0.002, *t*=2.57, and *P*=.01				
	Hopelessness (future thoughts)	—		Active SI: β=.02, SE 0.003, *t*=5.29, and *P*<.001				
	Pain tolerance	—		Suicidal behavior: β=.0001, SE 0.0002, *t*=0.30, and *P*=.76				
	Fearlessness about death	—		Suicidal behavior: β=.0001, SE 0.0002, *t*=0.46, and *P*=.64				
**Wolford** **-** **Clevenger et al [28], 2021**								SI
	Hopelessness (future thoughts)	—		b=0.26; SE 0.07; *t*=3.48; *P*<.001				

^a^SHTB: self-harm thought and behavior.

^b^SI: suicidal ideation.

^c^Standardized coefficient.

^d^No association reported.

^e^Unstandardized coefficient.

^f^OR: odds ratio.

**Table 6:** Summary of papers reporting associations between integrated motivational-volitional (IMV) model constructs and nonsuicidal self-harm thoughts and behaviors (SHTBs) in daily life.

Study and IMV construct measured			Concurrent associations		Lagged associations		SHTBs measured	
**Ammerman et al [50], 2017**								NSSI^a^
	Impulsivity	b^b^=0.62; SE 0.30; *P*<.05		—^c^				
**Burke et al [52], 2021**								NSSI urge
	Urgency (impulsivity)	—		b=1.39 (95% CI 0.78-2.00); *P*<.001				
**Christensen et al [53], 2023**								NSSI urge
	Social support	—		OR^d^ 0.49 (95% CI 0.32-0.78); *P*=.002				
**Czyz et al [45], 2019**								NSSI
	Engagement in coping strategies (number of strategies)	b=–0.08; SE 0.18; *P*=.67		—				
**Hughes et al [62], 2019**								NSSI thoughts and behaviors
	Rumination (repetitive negative thoughts)	—		NSSI thought intensity rating: β^e^=.01, *t*=6.75, and *P*<.001; NSSI behavior frequency: β=.03, *t*=7.73, and *P*<.001				
**Kellerman et al [30], 2022**								NSSI
	Social support from staff	—		AOR^f^ 0.67 (95% CI 0.50-0.90); *P*=.007				
	Social support from other patients	—		AOR 0.83 (95% CI 0.61-1.12); *P*=.23				
	Social support from family members	—		AOR 0.68 (95% CI 0.52-0.89); *P*=.005				
	Social support from friends	—		AOR 1.02 (95% CI 0.77-1.36); *P*=.87				
**Kudinova et al [65], 2023**								NSSI
	Humiliation	—		b=0.19; *P*>.05				
**Kuehn [66], 2021**								NSSI
	Impulsivity	OR 1.12 (95% CI 0.97-1.32)		—				
	Humiliation	—		OR 0.99 (95% CI 0.83-1.16)				
**Turner et al [33], 2016**								NSSI
	Perceived social support	—		When NSSI has been disclosed: OR 6.87 (95% CI 3.47-13.58), *t*=5.66, and *P*<.001; when NSSI has not been revealed: OR 0.73 (95% CI 0.34-1.54), *t*=–0.86, and *P*>.05; no NSSI: OR 1.05 (95% CI 0.94-1.17), *t*=0.83, and *P*>.05				
**Turner et al [40], 2019**								Intense NSSI urges
	Problem-focused coping	OR 0.92 (95% CI 0.83-1.03)		OR 0.91 (95% CI 0.79-1.04)				
	Avoidant coping	OR 1.15 (95% CI 1.03-1.29)		OR 0.96 (95% CI 0.84-1.10)				
	Perceived social support	OR 0.87 (95% CI 0.78-0.97)		OR 0.92 (95% CI 0.87-0.98; lagged, same day); OR 0.98 (95% CI 0.86-1.11; lagged, next day)				
**Victor et al [23], 2019**								NSSI urges
	Criticism (thwarted belongingness)	—		β=.02 (95% CI –.07 to .11)				
	Rejection (thwarted belongingness)	—		β=.20, (95% CI .11-.27)				

^a^NSSI: nonsuicidal self-injury.

^b^Unstandardized coefficient.

^c^No association reported.

^d^OR: odds ratio.

^e^Standardized coefficient.

^f^AOR: adjusted odds ratio.

#### Motivational Phase

The studies reported in 3% (2/58) of the papers measured associations between defeat and SI; one of them tested both concurrent and lagged associations [78], and one tested concurrent associations only [48]. Both papers reported evidence for a concurrent positive relationship between defeat and SI, but there was no evidence for a lagged association [78].

One paper [78] reported a significant positive association between entrapment and SI both concurrently (*P*<.001) and lagged (*P*=.01, 2-4 hour lag).

The studies reported in 3% (2/58) of the papers tested lagged associations between shame and NSSI [65] or suicidal thoughts [66]. Shame predicted increased suicidal thoughts but did not predict NSSI.

#### Threat-to-Self Moderators

The studies reported in 3% (2/58) of the papers measured associations between rumination and suicidal thoughts and behaviors; one of them tested both concurrent and lagged associations [73], and one tested lagged associations only [60]. One study tested lagged associations between rumination and NSSI thoughts and behaviors [62]. A significant association between rumination and SI or NSSI thoughts and behaviors was found in lagged models [60,62]. However, Rogers [73] found no evidence of concurrent or lagged associations between rumination or suicide-specific rumination and suicidal behaviors.

The studies reported in 9% (5/58) of the papers measured associations between different aspects of coping and suicidal thoughts and behaviors [43,66,76] or NSSI urges [40,45]. Of the 5 papers, 1 (20%) reported concurrent associations only, 2 (40%) reported lagged associations only, and 3 (60%) reported both. One paper reported evidence only of increased odds of NSSI urges for those reporting concurrent avoidant coping [40], and one reported negative lagged associations between engagement with positive activity-based and mindfulness-oriented coping techniques (distraction) and SI [76]. Kuehn [66] found that increased use of disengagement coping strategies predicted increased suicidal thoughts in concurrent but not lagged models.

#### Motivational Moderators

The studies reported in 11 papers measured associations between thwarted belongingness and suicidal thoughts or behaviors; 6 (55%) of these measured both concurrent and lagged associations, 1 (9%) measured concurrent associations only, and 4 (36%) measured lagged associations only.

A total of 12% (7/58) of the papers reported evidence of concurrent associations between thwarted belongingness and suicidal thoughts or behaviors [25,39,46,58], and 10% (6/58) reported evidence of lagged associations [39,58,59,63,79] (8/53, 15% of the studies found no evidence of lagged associations [23,25,46,56,73]). Wolford-Clevenger et al [79] found small but significant lagged associations with both passive and active SI. One paper additionally measured lagged associations between thwarted belongingness and NSSI urges [23]. Victor et al [23] found evidence of lagged associations with NSSI but not suicidal urges.

The studies reported in 9 papers measured associations between perceived burdensomeness and suicidal thoughts and behaviors, with all but 2 (22%) measuring both concurrent and lagged associations. Perceived burdensomeness predicted suicidal urge intensity [41] and SI [39,46,79] in both concurrent and lagged associations. In the study by Czyz et al [44], perceived burdensomeness was associated with SI frequency, duration, and urge severity in concurrent models but only with duration in lagged models. In the studies by Kleiman et al [25] and Gerner et al [58], perceived burdensomeness predicted SI in concurrent but not lagged models. The paper by Jacobucci et al [63] reported evidence of an association between perceived burdensomeness and SI in lagged models, and Rogers [73] found no evidence of concurrent or lagged associations with suicidal behaviors.

The studies reported in 11 papers measured associations between future thoughts and suicidal thoughts and behaviors; 6 (55%) of these tested both concurrent and lagged associations [25,37,44,46,58,73], 4 (36%) tested lagged associations only [28,29,51,79], and 1 (9%) tested concurrent associations only [48]. Future thoughts (operationalized in these studies as hopelessness) predicted SI in both concurrent and lagged models [28,29,41,46,48,58,79]. The study by Czyz et al [44] found evidence of concurrent (but not lagged) associations between hopelessness and SI frequency, duration, and urge severity. No lagged association was found between hopelessness and SI in 8% (4/53) of the studies [25,44,51,73].

The studies reported in 9% (5/58) of the papers measured associations between perceived social support and SI [54] or NSSI [30,33,40,53], with considerable variation in how social support was conceptualized. The paper by Turner et al [40] reported negative associations between perceived social support and NSSI urges in concurrent and lagged, same-day models but not in lagged, next-day models. In the paper by Turner et al [33], perceived social support following disclosure of NSSI positively predicted subsequent NSSI. Social support predicted a lagged decrease in NSSI urge in the paper by Christensen et al [53]. In a daily diary study of hospitalized adolescents, Kellerman et al [30] found decreased odds of NSSI in lagged models for social support from staff and family members but not from other patients or friends. Coppersmith et al [54] found negative concurrent—but not lagged—associations between social support and SI.

#### Volitional Moderators

The studies reported in 6 papers measured associations between impulsivity and SI [35,47,64,66] or NSSI [50,52,66]; 4 (67%) of these measured concurrent associations only [35,47,50,66], and 1 (17%) measured lagged associations only [52]. Ammerman et al [50] and Kaurin et al [47] found evidence of a positive concurrent association between impulsivity and NSSI or SI, respectively, and Burke et al [52] found evidence of a positive lagged association between impulsivity (conceptualized as urgency) and NSSI urges. However, 5% (3/58) of the papers [35,64,66] reported no evidence of a concurrent association between impulsivity (or impulsivity during social interactions [64]) and SI [35,64] or NSSI [66].

The studies reported in 3% (2/58) of the papers measured—and did not find evidence of—either concurrent or lagged associations between pain sensitivity and fearlessness about death and suicidal behavior [79] or SI [34].

Rogers [73] found evidence of a concurrent but not lagged association between “psychological access to means” and suicidal behaviors but no evidence of associations for fearlessness about death or physical access to means. Bayliss et al [34] did not find evidence of either concurrent or lagged associations between access to means and SI.

One paper [34] reported both concurrent and lagged associations between mental imagery and SI.

### Quality of Evidence

As there are currently no gold standards for conducting EMA research, we assessed the studies against quality criteria for *reporting* EMA studies. The reporting quality of the studies varied considerably, although most were fully compliant with only a small minority of the quality criteria. The reporting criteria with the most compliance included referencing EMA (or equivalent) in the title and keywords, briefly introducing and justifying the use of EMA, reporting the full text of the items and response options, and describing data preparation and analysis in detail (Table 7). The criteria with the poorest compliance included justifying the sample size, justifying the sample design (eg, random or event based) and number of assessments, describing any design feature to address potential sources of bias or participant burden (eg, EMA questions appearing in different orders), reporting the number of EMA prompts that were planned to be delivered and the number that was actually received by participants (and any reasons why prompts were not sent out), and reporting whether EMA compliance was related to demographic or time-varying variables.

No study fully reported the amount of time from prompt signal to answering of the prompt. There was generally low use of open science practices, with only the studies reported in 3% (2/58) of the papers explicitly preregistering hypotheses and 2% (1/58) publishing study materials. The studies reported in 14% (8/58) of the papers made their analytic code either fully or partially publicly available, and those reported in 7% (4/58) of the papers made their data open access (other studies either reported data being available on request or did not include a data availability statement). However, it is worth noting that such open science practices are recent responses to the replication crisis and would not be expected in papers published before 2016. A detailed summary table of quality assessment data is available in Multimedia Appendix 4.

**Table 7:** Reporting quality assessment summary (N=58)^a^.

Section and reporting criterion			Papers with full compliance, n (%)
**Title and abstract**			
	Included EMA^b^ (or equivalent) in title and keywords	34 (59)	
**Introduction**			
	Briefly introduced the concept of EMA and the reasons for using it	41 (71)	
**Methods**			
	Detailed training of participants for the EMA protocol	19 (33)	
	Described procedures used to enhance compliance and participation	35 (60)	
	Described the technology used (hardware and software)	29 (50)	
	Justified the sample size	10 (17)	
	Explained the rationale for sampling density (assessments per day) and scheduling	14 (24)	
	Reported the full text of the items, rating time frames, response options, and scaling	35 (60)	
	Reported psychometric properties and origins of items	15 (26)	
	Preparation for data analyses—described centering of predictor variables and at what level; reported covariates included in the models	36 (62)	
	Described levels of analysis (momentary, day, and person level); described modeling and statistical software used	44 (76)	
**Results**			
	Described the final dataset—number of reports (total, person average, and group average), days in study and retention rates, and rates of delayed or suspended responding	18 (31)	
	Reported the amount of time from prompt signal to answering of prompt	0 (0)	
	Reported compliance rate by monitoring both days and waves, if applicable; indicated reasons for noncompliance, if known	8 (14)	
	Reported whether EMA compliance was related to demographic or time-varying variables	9 (16)	
**Discussion**			
	Discussed EMA-specific limitations of the study (eg, reactivity, use of technology, use of unvalidated measures, software or hardware limitations, and compliance)	19 (33)	
**Transparency and reproducibility**			
	A locked version of the hypotheses, research questions, analysis plan, or methods was registered before data access and analysis	2 (3)	
	Materials (eg, full EMA questionnaire, any other questionnaires, and instructions to participants) were available	1 (2)	
	Code used to conduct the analysis was available online	6 (10)	
	Data were publicly available or stored in a restricted-access repository	4 (7)	

^a^The full criteria are available in Multimedia Appendix 4 [22-79].

^b^EMA: ecological momentary assessment.

## Discussion

### Principal Findings

There has been a recent proliferation in EMA studies of SHTBs. We set out to summarize the state of the EMA literature pertaining to the constructs described in the IMV model. We selected the IMV model because it is one of the leading theoretical frameworks for understanding suicidal behavior that incorporates most constructs contained in other theoretical models, such as the interpersonal theory of suicide (IPTS) [80].

A total of 58 papers were included in this review. The IMV constructs measured most frequently were thwarted belongingness (24/58, 41% of the papers), thoughts about the future (20/58, 34% of the papers), and perceived burdensomeness (16/58, 28% of the papers). The higher number of studies focusing on thwarted belongingness and perceived burdensomeness likely reflects the key role of these constructs in the IPTS [80]. The least frequently measured constructs were humiliation, social problem-solving, mental imagery, physical pain sensitivity, and fearlessness about death. No included papers measured memory biases, goals, norms, or resilience using EMA.

Most of the included papers were assessed to be fully compliant with a number of reporting quality criteria, including referencing EMA (or equivalent) in the title and keywords (34/58, 59%), briefly introducing and justifying the use of EMA (41/58, 71%), reporting the full text of the items and response options (35/58, 60%), and describing data preparation and data analysis in detail (36/58, 62%). However, there was poor compliance with over half of the 25 criteria against which the reporting quality of the included papers was assessed.

The results predominantly showed at least moderate within-person variability across all IMV constructs included in this review whether assessed once or multiple times daily, highlighting the utility of intensive longitudinal study designs. Moderate to high within-person variability characterizes these constructs as at least somewhat statelike and dynamic. Where shown to be associated with SHTBs, these cognitive, social, and situational stressors and coping resources outlined in the IMV model could be important targets for intervention. Finding statelike constructs can identify risk factors that are potentially suitable for in-the-moment or just-in-time interventions before SHTBs develop. Although some constructs from the IMV model were well researched using EMA, namely, thwarted belongingness, future thoughts, and perceived burdensomeness, less—or no—information was available for other constructs, such as defeat, humiliation, social problem-solving, memory biases, resilience, access to means, fearlessness about death, and exposure to suicide. Some of these (social problem-solving, memory biases, and resilience) are likely to be more traitlike constructs and, thus, less suited to EMA research.

We did not find evidence of patterns in within-person variability as a function of whether the samples were drawn from community or clinical populations or of the number of daily assessments. It is worth noting that most community samples were “high-risk” populations with a recent history of SHTBs who may not have differed substantially from clinical populations. Our ability to make comparisons between sampling schemes was also limited by a quarter of the included studies (13/58, 22%) not reporting within-person variance.

We found evidence across several studies (15/58, 26%) of concurrent associations between IMV constructs and SHTBs and some—though inconsistent—evidence of lagged associations between entrapment and SI, shame and SI, rumination and SHTBs, future thoughts and SHTBs, perceived burdensomeness and SHTBs, impulsivity and NSSI, thwarted belongingness and SHTBs, social support and NSSI, and mental imagery and SI. However, not all findings were consistent, possibly due to heterogeneity across the studies included in our review in terms of the populations studied, study design, operationalization of both IMV constructs and SHTBs, and analytical approach. While the cross-lagged panel model—sometimes used in the included studies—is a popular tool in this and other fields, it may often not be the correct tool [81]. The importance of disaggregation when analyzing EMA data to study within-person processes has been recognized, and we are likely to see a continued transition to alternative, superior models, such as the random-intercepts cross-lagged panel model. Failure to disaggregate within- and between-person variability is likely to lead to much higher lagged effects, particularly when studying stable, traitlike constructs.

Inconsistencies in concurrent and lagged associations between IMV constructs and SHTBs preclude firm conclusions about support for these constructs as proximal risk factors for SHTBs. We did not identify studies explicitly testing hypotheses from the IMV model, namely, moderation of the association among defeat, humiliation, and entrapment; between entrapment and SI; or between SI and suicidal behavior. Our review shows that there is scope for further investigation of temporal relationships between IMV constructs and SHTBs, particularly with regard to the motivational phase (defeat, humiliation, and entrapment), threat-to-self moderators (social problem-solving, coping, memory biases, and rumination), and volitional moderators (access to means, planning, impulsivity, physical pain sensitivity, fearlessness about death, and imagery) in the model. While our review suggests that most IMV constructs are subject to momentary shifts, more evidence is needed as to the implications of these dynamic patterns for SHTBs and progression from SI to behavior.

Assessment of the reporting quality of the included studies suggested little routine use of open science principles—even in studies published since the replication crisis—reflecting the situation in the suicide research field more broadly [82]. Open science practices can improve the transparency, reproducibility, and replicability of scientific research [83]. We recommend that future EMA studies in the field consider preregistration of hypotheses using the registration template for ESM research [82] and making data, study materials, and analytic code available. Other key areas for improvement include justifying the sample size (eg, by reporting power analyses [84]), sample design, and the number of assessments used in a study; consideration of potential sources of bias or participant burden when designing EMA studies; and more comprehensive reporting of EMA compliance, including whether compliance is related to demographic or time-varying variables. This is crucial as aspects of EMA study design, such as great questionnaire length, are associated with increased burden, which affects the quality and quantity of EMA data [85].

### Comparison With Prior Work

Our findings are broadly consistent with a recent systematic review of interpersonal processes and SHTBs in daily life [18] in which the authors highlight a lack of consistency in the operationalization of key constructs [16]. Efforts to improve transparency and harmonization of measurement include the ESM Item Repository [86], a searchable and public bank of items used in previous EMA studies to which researchers can contribute their items.

Also consistent with previous reviews of the EMA SHTB literature [8,14,16,18], our findings indicate that EMA is a useful methodological approach to providing rich information about individuals’ experiences of both IMV constructs and SHTBs in daily life. The same reviews have similarly noted the challenges to synthesis of findings due to heterogeneity in study design. Despite the wide variation in results reported, we found evidence of at least moderate within-person variance in IMV constructs. This adds to existing EMA literature reporting SHTBs to be subject to short-term fluctuation [8,13]. While SHTBs have been well studied using EMA methodology, risk factors in the IMV model have received less attention. EMA is particularly relevant to the IMV model of suicide, enabling an ecologically valid “in-situ” and nuanced understanding of an individual’s transition through the motivational and volitional phases of the model, including identification of immediate situational triggers and critical moments of escalation from ideation to behavior [3,9].

Our findings of mixed evidence of associations between IMV constructs and SHTBs are also broadly consistent a review of (mainly cross-sectional) studies testing hypotheses from the IPTS [87], which found mixed support for a main effect of thwarted belongingness on SI, with stronger—though still inconsistent—support for a main effect of perceived burdensomeness on SI. Also in line with our findings, the aforementioned review [87] found lower support for an association between acquired capability for suicide (including pain sensitivity and fearlessness about death) and suicide attempt.

### Strengths and Limitations

Our review is the first to comprehensively synthesize the EMA literature on key constructs in the IMV model of suicide [3], including a narrative synthesis of both within-person variance of constructs and their associations with SHTBs. It also adds to the findings from a recent systematic review of cross-sectional, case-control, and longitudinal studies of the IMV model [5]. The broad scope of this review, in addition to the methodological differences between studies, prohibited a meta-analysis of findings. Future meta-analyses of variability in key IMV constructs may be feasible with more focused and specific inclusion criteria.

We conducted a thorough search of 5 databases as well as citation searching and used blinded double screening of abstracts against the inclusion and exclusion criteria. We made extra efforts to include unpublished research by searching the PMC Europe database of preprints, therefore limiting the effect of possible publication bias on our results. An additional search in 3 months of submission for publication ensured that the review was as up-to-date as possible as the EMA literature is prolific. We limited the search to papers in English following consultation with colleagues but acknowledge that this may have resulted in the loss of papers published in other languages.

### Implications

Our findings suggest that EMA methods can be valuable in providing real-time information about key risk factors for SHTBs, as outlined in the IMV model [3]. EMA may also be of potential value in clinical support settings, providing clinicians with detailed insights into an individual’s mental states and daily experiences of constructs described by the IMV model. Enhanced understanding of these risk factors in daily life may inform individualized interventions.

### Future Research

Further research is needed to better understand how some IMV constructs—including defeat, humiliation, social problem-solving, memory biases, resilience, access to means, fearlessness about death, and exposure to suicide—vary in daily life and over what timescale and whether there are proximal associations with SHTBs. Many of the factors we investigated are conceptualized in the IMV model as moderators; however, all studies in this review focused on individual relationships with SHTBs. Further research is needed to explore moderation to specifically test the hypotheses outlined in the IMV model.

We found large heterogeneity in the populations sampled, including gender (eg, 2/53, 4% of the studies sampled only female participants [22,23], and 1/53, 2% of the studies sampled only transgender or gender-diverse participants [28]), ethnicity (eg, 1/53, 2% of the studies sampled only Hispanic or Latino adults [75]), and mental health status (40/53, 75% of the studies sampled only those with a diagnosed mental health disorder or with recent experience of SHTBs). Female participants were overrepresented in EMA studies, representing 69.8% of the total combined sample. While beyond the scope of this review, future EMA studies or reviews might consider sociodemographic differences in the within-person variability of IMV constructs.

### Conclusions

Overall, there is existing evidence suggesting that there is within-person fluctuation in the IMV constructs included in this review, suggesting that it is possible to study them using EMA methods. We also found evidence of concurrent relationships between almost all constructs and SHTBs in daily life, with some evidence that entrapment, shame, rumination, thwarted belongingness, hopelessness, social support, and impulsivity are additionally associated with SHTBs in lagged (ie, longitudinal) relationships. While EMA methods show promise in providing valuable information about individuals’ experiences of both IMV constructs and SHTBs in daily life, there is currently large methodological heterogeneity and a paucity of quality in studies using this approach. Efforts to enhance the quality of reporting and advance transparency and harmonization in the field may improve future syntheses of findings.

## Appendix

Multimedia Appendix 1 PRISMA (Preferred Reporting Items for Systematic Reviews and Meta-Analyses) checklist.

Multimedia Appendix 2 Proportion of within-person variance reported for each integrated motivational-volitional model construct by sample type and number of assessments per day.

Multimedia Appendix 3 Overview of the included studies with item wording.

Multimedia Appendix 4 Reporting quality assessment summary.

## Abbreviations

EMA: ecological momentary assessment
ESM: experience sampling method
ICC: intraclass correlation coefficient
IMV: integrated motivational-volitional model
IPTS: interpersonal theory of suicide
NSSI: nonsuicidal self-injury
OSF: Open Science Framework
PRISMA: Preferred Reporting Items for Systematic Reviews and Meta-Analyses
SHTB: self-harm thought and behavior
SI: suicidal ideation
