# Supplementary material for: Ecological Momentary Assessment of Self-Harm Thoughts and Behaviors: Systematic Review of Constructs From the Integrated Motivational-Volitional Model
Source: JMIR Ment Health. 2024 Dec 9;11:e63132. doi: 10.2196/63132 (PMC11667137; doi:10.2196/63132)
Supplement: Multimedia Appendix 2 [file mental_v11i1e63132_app2.docx]

Multimedia Appendix 2

Table S2 Proportion of within-person variance reported for each IMV construct, by sample type and number of assessments per day.^[[1]](#footnote-1)^

|  | **1x per day (daily diary)** | | **2-4x per day** | | **5-10x per day** | | **Range across all studies**^[[2]](#footnote-2)^ | |
| --- | --- | --- | --- | --- | --- | --- | --- | --- |
|  | **Clinical sample** | **Community sample** | **Clinical sample** | **Community sample** | **Clinical sample** | **Community sample** | **Clinical sample** | **Community sample** |
| **Motivational phase** | | | | | | | | |
| Defeat |  |  |  |  | **53%** (mixed sample; van Ballegooijen et al., 2022) | **48%** (Stenzel et al., 2020) | **53% (1)** | **48% (1)** |
| Shame (humiliation) |  |  |  |  | **89%** (Kudinova et al., 2023)  **62%** (mixed sample; Kuehn, 2021)  **18%** (Bentley et al., 2021) |  | **18-89% (3)** | **-** |
| Entrapment |  | **63%** (Cloos et al., 2020) | **48%** (Nuij et al., 2022) |  | **39%** (mixed sample; van Ballegooijen et al., 2022) | **46%** (Stenzel et al., 2020) | **39-48% (2)** | **46-63% (2)** |
| **Threat-to-self moderators** | | | | | | | | |
| Coping | **49-73%** (Al-Dajani et al., 2022) | **28-42%** (Ewing & Hamza, 2024) | **52%** (Czyz et al., 2023a) |  | **68-96%** (mixed sample; Kuehn, 2021) |  | **49-96% (3)** | **28-42% (1)** |
| Rumination | **53%** (Czyz et al., 2021) | **78%** (Mitchell et al., 2023) | **53%** (Nuij et al., 2022)  **41%** (Czyz et al., 2023a) |  | **84%** (mixed sample; Kuehn, 2021)  **53%** (mixed sample; van Ballegooijen et al., 2022) | **50%** (Hughes et al., 2019)  **44%** (Rogers, 2023)  **20%** (suicide specific; Rogers, 2023) | **41-84% (5)** | **20-78% (4)** |
| Social problem-solving |  |  | **34%** (Nuij et al., 2022) |  | **75%** (mixed sample; Kuehn, 2021) |  | **34-75% (2)** | **-** |
| **Motivational moderators** | | | | | | | | |
| Future thoughts | **33%** (Czyz et al., 2019a)  **42%** (Czyz et al., 2021)  **37%** (Tsypes et al., 2022) | **70%** (Kirtley et al., 2022) | **27%** (Czyz et al., 2023a)  **34%** (Kleiman et al., 2017)  **40%** (Nuij et al., 2022)  **56%** (Nuij et al., 2022) | **51%** (Al-Dajani & Uliaszek, 2021)  **43%** (Kleiman et al., 2017) | **26%** (Hallensleben et al., 2019);  **55%** (Krall et al., 2024)  **52%** (Reeves, 2022)  **52%** (mixed sample; van Ballegooijen et al., 2022) | **63%** (Gerner et al., 2023);  **22%** (Krall et al., 2024)  **37%** (Rogers, 2023) | **26-56% (11)** | **22-70% (6)** |
| Perceived burdensomeness | **60%** (Al-Dajani & Czyz, 2022)  **31%** (Czyz et al., 2019a)  **38%** (Czyz et al., 2021)  **33%** (mixed sample; MacNeil et al., 2023) |  | **29%** (Czyz et al., 2023a)  **31%** (Parrish et al., 2021)  **19%** (Silva et al., 2022)  **14%** (Nuij et al., 2022) |  | **34%** (Hallensleben et al., 2019)  **42%** (Kleiman et al., 2017)  **56%** (Reeves, 2022) | **47%** (Gerner et al., 2023)  **37%** (Rogers, 2023) | **14-60% (11)** | **37-47% (2)** |
| (Thwarted) belongingness | **57%** (family) **61%** (peer) (Al-Dajani & Czyz, 2022)  **37% (**Czyz et al., 2019a)  **56%** (friends)  **41%** (family) (Czyz et al., 2021) | **47%** (mixed sample; MacNeil et al., 2023)  **33%** (Molaie, 2022) | **41%** (Czyz et al., 2023a)  **22-33%** (Glenn et al., 2022)  **41%** (Parrish et al., 2021)  **44%** (Peters et al., 2022)  **15%** (Silva et al., 2022)  **4%** (Silva et al., 2022) | **50%** (Harper, 2019)  **39%** (Kleiman et al., 2017) | **43%** (Hallensleben et al., 2019)  **51%** (Kleiman et al., 2017)  **26%** (Reeves, 2022)  **42%** (mixed sample; van Ballegooijen et al., 2022) | **36%** (Gerner et al., 2023)  **61%** (Lopez et al., 2023)  **46%** (Rogers, 2023)  **90%** (Victor et al., 2019)  **83%** (Victor et al., 2019) | **4-57% (15)** | **33-90% (9)** |
| Social support | **29%** (hospital staff);  **27%** (other patients);  **26%** (family);  **19%** (friends) (Kellerman et al., 2022) | **56%** (Coppersmith et al., 2019)  **44%** (Turner et al., 2016) |  |  | **98%** (mixed sample; Kuehn, 2022) | **22%** (Christensen et al., 2023) | **19-98% (5)** | **22-56% (3)** |
| **Volitional moderators** | | | | | | | | |
| Impulsivity | **64-78%** (Lucht et al., 2022)  **25%** (Nuij et al., 2022) | **59%** (Jeong et al., 2021) | **46%** ( Kaurin et al., 2022) |  | **25%** (mixed sample; Kuehn, 2022) |  | **25-78% (4)** | **59% (1)** |
| Mental imagery |  | **56-79%** (Cloos et al., 2020) | **64%** (Nuij et al., 2022) | **72%** (Bayliss et al., 2024) |  |  | **64% (1)** | **56-79% (2)** |
| Physical pain sensitivity | **48%** (Spangenberg et al., 2019) | **61%** (Krall et al., 2024) |  | **29%** (Bayliss et al., 2024) | **74%** (mixed sample; Selby et al., 2019)  **76%** (Krall et al., 2024) |  | **48-74% (3)** | **29-61% (2)** |
| Fearlessness about death | **53%** (Spangenberg et al., 2019) |  |  | **31%** (Bayliss et al., 2024) |  | **12%** (Rogers, 2023) | **53% (1)** | **12-31% (2)** |
| Access to means |  |  |  | **45%** (Bayliss et al., 2024) |  | **34%** (Rogers, 2023) | **-** | **34-45% (2)** |

1. Within-person variance = 1- ICC [↑](#footnote-ref-1)
2. Number of studies indicated in parentheses. [↑](#footnote-ref-2)
