# Supplementary material for: Ecological Momentary Assessment of Self-Harm Thoughts and Behaviors: Systematic Review of Constructs From the Integrated Motivational-Volitional Model
Source: JMIR Ment Health. 2024 Dec 9;11:e63132. doi: 10.2196/63132 (PMC11667137; doi:10.2196/63132)
Supplement: Multimedia Appendix 3 [file mental_v11i1e63132_app3.docx]

Multimedia Appendix 3

Table S3 Overview of included studies with item wording

| **Authors** | **Sample type** | **Sample size** | **Study duration, days** | **Number of assessments per day** | **Constructs measured** | **Item wording or scale used** | **ICC reported** |
| --- | --- | --- | --- | --- | --- | --- | --- |
| Aadahl et al., 2021 [48] | Mixed (clinical and community) | 27 | 7 | 6 | Defeat | Right now… I feel powerless; …I am one of life's losers | - |
|  |  |  |  |  | Entrapment | I am trapped in my situation; There are things in my life I want to escape | - |
|  |  |  |  |  | Hopelessness | I look forward to the future; Things don't work out the way I want | - |
|  |  |  |  |  | Suicidal ideation | Right now… I want to die; Just before the text… I was thinking about killing myself | n/a |
| Al-Dajani & Czyz, 2022 [41] | Clinical (inpatient) | 78 | 28 | 1 | Perceived burdensomeness | I felt people in my life would be happier without me | 0.40 |
|  |  |  |  |  | Peer belongingness | I felt close to my friends | 0.59 |
|  |  |  |  |  | Family belongingness | I felt close to my family | 0.43 |
|  |  |  |  |  | Suicidal urge intensity | How strong was the urge to act on your thoughts of suicide? | n/a |
| Al-Dajani & Uliaszek, 2021[49] | Mixed clinical and non-clinical | 39 | 14 | 4 | Hopelessness | Rate how hopeless you feel right now | 0.49 |
| Al-Dajani et al., 2022 [43] | See Al-Dajani & Czyz, 2022 | | | | Coping (personal-support) | (1) talked with a friend or peer, (2) talked with a parent or family member | 0.49 |
|  |  |  |  |  | Coping (professional-support) | (3) contacted a crisis line (call, text, or chat, (4) talked with a therapist, counselor, or doctor | 0.27 |
|  |  |  |  |  | Coping (non-cognitive) | (5) did something relaxing or comforting, (6) distracted self with something else | 0.38 |
|  |  |  |  |  | Coping (cognitive) | (7) tried to tell self something calming or positive, and (8) either (a) thought about reasons for living (on days when SI endorsed) or (b) thought about something that makes self feel better (on days without SI) | 0.55 |
|  |  |  |  |  | Coping (perceived helpfulness) | - | 0.42 |
|  |  |  |  |  | Coping (total strategies used) | - | 0.51 |
|  |  |  |  |  | Suicidal urge intensity | How strong was the urge to act on your thoughts of suicide? | n/a |
| Ammerman et al., 2017 [50] | Non-clinical | 51 | 7 | 4 | Impulsivity | To what extent do you feel this way, right now ... Impulsive | - |
|  |  |  |  |  | NSSI | Participants were asked if, since the last phone call, they had engaged in four different forms of self-injury (cutting, burning, poking/biting, and banging head), in addition to any ''other'' self-injurious behavior, without the intent to die | n/a |
| Baryshnikov et al., 2024 [29] | Clinical (inpatient) | 67 | Varied (M=3.4 days) | 3 | Hopelessness | - | - |
|  |  |  |  |  | Suicidality | - | n/a |
| Bayliss et al., 2024 [34] | Non-clinical | 75 | 14 | 4 | Mental imagery | In the last 15 min “I have had mental images of myself planning/preparing to harm myself or make a suicide attempt” | 0.28 |
|  |  |  |  |  | Fearlessness about death | In the last 15 min “I have not been afraid of death | 0.69 |
|  |  |  |  |  | Pain sensitivity | In the last 15 min “I could handle pain more easily than other people” | 0.71 |
|  |  |  |  |  | Access to means | In the last 15 min “I could access the method/means I would use to kill myself” | 0.55 |
|  |  |  |  |  | Suicidal ideation | I want to die by suicide | n/a |
| Bentley et al., 2021 [24] | Clinical (inpatient) | 83 | Varied (M =8.52 days, SD=5.73; range: 2-46) | 4 | Shame (humiliation) | Participants were asked to rate how much they were currently feeling: ashamed, guilty, humiliated, and self-hate (“shame/selfhatred”) | 0.82 |
| Ben-Zeev et al., 2012 [51] | Clinical (inpatient) | 31 | 7 | 6 | Helplessness | - | - |
|  |  |  |  |  | Hopelessness | Are you discouraged about the future? | - |
|  |  |  |  |  | Suicidal ideation | Since you were last beeped have you had any thoughts of killing yourself? | n/a |
| Burke et al., 2021 [52] | University | 60 | 10 | 3 | Impulsivity | Right now… I feel like doing something I will later regret in order to make myself feel better now,” “… it feels hard to resist acting on my feelings,” and “… it feels hard to keep my feelings under control.” | - |
|  |  |  |  |  | NSSI urge | “Right now, how intense is your urge to engage in non-suicidal self-injury?” | n/a |
| Christensen et al., 2023 [53] | Non-clinical | 93 | 7-14 | 6 | Social support | At this moment... ...I have someone who understands my problems ...I feel there are people I can talk to if I am upset. | 0.78 |
|  |  |  |  |  | NSSI urge | Since the last survey you completed... Did you have the urge to hurt yourself on purpose, but not to kill yourself? | n/a |
| Cloos et al., 2020 [22] | University | 19 | 10 | 1 | Entrapment | How much did you feel entrapped in your current situation today? Meaning that you perceived your current situation as unpleasant (e.g., due to own thoughts or feelings, or to outer circumstances) and would have liked to leave it yet felt unable to. | 0.37 |
|  |  |  |  |  | Mental imagery Compellingness | Self-harm Images Interview (SHII, 20 items) | 0.21 |
|  |  |  |  |  | Mental imagery Vividness |  | 0.29 |
|  |  |  |  |  | Mental imagery Controllability |  | 0.22 |
|  |  |  |  |  | Mental imagery Nowness |  | 0.29 |
|  |  |  |  |  | Mental imagery Distress |  | 0.33 |
|  |  |  |  |  | Mental imagery Comfort |  | 0.44 |
| Coppersmith et al., 2019 [54] | Non-clinical | 53 | 28 | 1 | Social support | Participants asked to rate how supported they felt from friends and family (in two separate items) that day compared to a typical day | 0.44 |
|  |  |  |  |  | Suicidal ideation | (1) wish to live, (2) wish to die, and (3) desire to die by suicide on a three point scale (moderate to strong, weak, none) | n/a |
| Czyz et al., 2019a [44] | Clinical (inpatient) | 34 | 28 | 1 | Hopelessness | I see only bad things ahead of me, not good things | 0.67 |
|  |  |  |  |  | Perceived burdensomeness | The people in my life would be happier without me | 0.69 |
|  |  |  |  |  | Connectedness | I am close to other people | 0.63 |
|  |  |  |  |  | Suicidal ideation | At any point in the last 24 hr, did you have any thoughts of killing yourself? | n/a |
| Czyz et al., 2019b [45] | See Czyz et al., 2019a | | | | Coping (number of strategies used) | When you had thoughts of killing yourself in the last 24 h, did you do any of these things to deal or cope with your thoughts? | - |
|  |  |  |  |  | NSSI | At any point in the last 24 h, did you harm yourself or hurt your body on purpose (such as cutting/burning your skin, or hitting yourself) without the intention to die? | n/a |
| Czyz et al., 2021 [42] | See Al-Dajani & Czyz, 2022 | | | | Hopelessness | I see only bad things ahead of me, not good things | 0.58 |
|  |  |  |  |  | Perceived burdensomeness | The people in my life would be happier without me | 0.62 |
|  |  |  |  |  | Connectedness to friends | I feel close to my friends | 0.44 |
|  |  |  |  |  | Connectedness to family | I feel close to my family | 0.59 |
|  |  |  |  |  | Rumination | I was dwelling on my feelings and problems | 0.47 |
|  |  |  |  |  | Suicidal ideation | How many times did you have thoughts of killing yourself? | n/a |
| Czyz et al., 2023 [41] | Clinical (outpatient) | 102 | 56 days | 4 | Rumination | I was dwelling on my feelings and problems | 0.59 |
|  |  |  |  |  | Hopelessness | I see only bad things ahead of me not good things | 0.73 |
|  |  |  |  |  | Perceived burdensomeness | I felt people in my life would be happier without me | 0.71 |
|  |  |  |  |  | Closeness to others | I felt close to my family | 0.59 |
|  |  |  |  |  | Coping | Today, how much did you do these things to deal or cope with your feelings or any stressful situations? [includes cognitive, non-cognitive, and support-seeking strategies] | 0.48 |
|  |  |  |  |  | Self-harm, suicidal ideation | Thinking about yesterday how many times did you harm yourself or hurt your body on purpose (such as cutting, burning, biting, hitting self) without the intention to die; Frequency of death thoughts (0-4) Evening EMA survey Today, how many times did you wish you were dead or that you could go to sleep and not wake up? Frequency of suicidal ideation (0-4) Evening EMA survey Today, how many times did you have thoughts of killing yourself? Intensity of suicidal ideation (0-5) Evening EMA survey Today, how strong was your intention to kill yourself? | n/a |
| Defayette et al., 2023 [56] | University | 42 | 28 | 6 | Thwarted belongingness (social exclusion) | How strongly do you feel excluded? | - |
|  |  |  |  |  |  |  |  |
|  |  |  |  |  | Suicidal ideation | - | n/a |
| Ewing & Hamza, 2024 [57] | University | 160 | 14 | 1 | Coping (problem-focused) | Students were asked to indicate how they responded to stressful life events they experienced the previous day using the Brief COPE | 0.58 |
|  |  |  |  |  | Coping (avoidant) |  | 0.63 |
|  |  |  |  |  | Coping (emotion-focused) |  | 0.72 |
|  |  |  |  |  | Coping (socially supported) |  | 0.63 |
| Gerner et al., 2023 [58] | University | 43 | 10 | 5 | Thwarted belongingness | I expect that people will never care about me | 0.64 |
|  |  |  |  |  | Perceived burdensomeness | I believe I will always fail the people in my life | 0.53 |
|  |  |  |  |  | Hopelessness | How hopeless do you feel right now? | 0.37 |
|  |  |  |  |  | Suicidal ideation | How intense is your desire to kill yourself right now? | n/a |
|  |  |  |  |  | Suicidal ideation | How intense is your desire to kill yourself right now? | n/a |
| Glenn et al., 2022 [59] | Clinical (outpatient) | 48 | 28 | 3 | Thwarted belongingness | Family [friend] belongingness items (items scored so higher values indicate greater thwarted belonging)   1. My family [friends] cares about me (reverse scored) 2. I feel disconnected from my family [friends] 3. I feel that I can turn to my family [friends] in times of need (reverse scored)   I am close to my family [friends] (reverse scored) | 8 items ranging from 0.67-0.78 |
|  |  |  |  |  | Suicidal ideation | How intense is your desire to kill yourself right now? | n/a |
| Hallard et al., 2021 [60] | Clinical (inpatient) and non-clinical | 24 | 6 | 7 | Rumination | Just before the text.. … I was thinking about my failures and weaknesses; I was analysing why I feel the way I do | - |
|  |  |  |  |  | Suicidal ideation | Right now.. I want to die. Just before the text… I was thinking about killing myself | n/a |
| Hallensleben et al., 2019 [46] | Clinical (inpatient) | 79 | 6 | 10 | Hopelessness | My future seems dark to me., I might as well give up because there is nothing I can do about making things better for myself. | 0.74 |
|  |  |  |  |  | Perceived burdensomeness | I feel useless., …like a burden for others. | 0.66 |
|  |  |  |  |  | Thwarted belongingness | I feel lonely., …like I do not belong | 0.57 |
|  |  |  |  |  | Passive and active suicidal ideation | passive (Life is not worth living for me.; There are more reasons to die than to live for me.) and active SI (I think about taking my life., I want to die.) | n/a |
| Harper, 2019 [61] | University | 145 | 7 | 3 | Loneliness (thwarted belongingness) | Six items from the UCLA Loneliness scale, “I could not find companionship when I wanted it”; “I felt like I didn’t belong”; “I felt like no one really knows me”; and “I felt lonely”. | 0.5 |
| Hughes et al., 2019 [62] | Non-clinical | 47 | 14 | 5 | Rumination | “I am experiencing many thoughts that are repeating over and over,” “I am experiencing many repetitive thoughts about how I am currently feeling,” “My thoughts are flowing from one thought to the next more quickly than they usually do,” and “I am experiencing many thoughts that are difficult for me to control or change.” | 0.7 |
|  |  |  |  |  | NSSI thoughts and behaviours | Participants were asked whether they had had thoughts of NSSI or engaged in any NSSI behaviours (yes/no) since the previous entry | n/a |
| Jacobucci et al., 2023 [63] | Non-clinical | 35 | 30 | 4 | Perceived burdensomeness | I feel like a burden | - |
|  |  |  |  |  | Thwarted belongingness | I feel like I do not belong | - |
|  |  |  |  |  | Suicidal ideation | “I think about taking my life” | - |
| Jeong et al., 2021 [27] | Non-clinical | 23 | 15 | 1 | Impulsivity | 12 items from UPPS-P Impulsive Behavior Scale | 0.41 |
|  |  |  |  |  | Suicidal ideation | - | n/a |
| Kaurin et al., 2022 [64] | Clinical and non-clinical | 186 | 21 | Event-contingent | Impulsivity (during a social interaction) | How would you describe your behavior during the interaction? (0: in control – 100: impulsive) | 0.54 |
|  |  |  |  |  | Suicidal ideation | “Since the interaction,” “Have you wished you were dead or wished you could go to sleep and not wake up?” and “Have you actually had any thoughts of killing yourself?” | n/a |
| Kaurin et al., 2023 [47] | Clinical and non-clinical | 153 | 21 | 6 | Impulsivity | Irresponsibility (“I said/did things that I wish I hadn’t”), non-planfulness (“I acted without thinking”), and risk taking (“I did something risky”) | - |
|  |  |  |  |  | Suicidal ideation | “Have you wished you were dead or wished you could go to sleep and not wake up?” and “Have you actually had any thoughts of killing yourself?” | n/a |
| Kellerman et al., 2022 [30] | Clinical (inpatient) | 118 | Varied (M=6.1, SD=6.1) | 1 | Social support from staff | Since the last check-in, [my friends/ my family members/ etc] have been…0: most unsupportive – 10: most supportive | 0.71 |
|  |  |  |  |  | Social support from other patients |  | 0.73 |
|  |  |  |  |  | Social support from family members |  | 0.74 |
|  |  |  |  |  | Social support from friends |  | 0.81 |
|  |  |  |  |  | NSSI | Have you hurt yourself on purpose since the last check in? | n/a |
| Kirtley et al., 2022 [38] | Non-clinical | 743 | 6 | 10 | Short-term future thinking (1x per day) | How much are you looking forward to today? | 0.3 |
| Kleiman et al., 2017 [25] | Non-clinical | 54 | 28 | 4 | Hopelessness | How much do you feel..[hopeless/ lonely/ burdensome]? | 0.57 |
|  |  |  |  |  | Loneliness |  | 0.49 |
|  |  |  |  |  | Perceived burdensomeness |  | 0.58 |
|  |  |  |  |  | Suicidal ideation | How intense is your desire to kill yourself right now?, How strong is your intention to kill yourself right now?, How strong is your ability to resist the urge to kill yourself right now? | n/a |
|  | Clinical (inpatient) | 36 | Varied (M=10.3, SD=6.5) | 4 | Hopelessness | How much do you feel..[hopeless/ lonely]? | 0.66 |
|  |  |  |  |  | Loneliness |  | 0.61 |
|  |  |  |  |  | Suicidal ideation | See above | n/a |
| Krall et al., 2024 [31] | University | 129 | 56 | 7 | Pain (1x per day) | Over the course of the day, how much did you feel physical pain? | 0.39 |
|  |  |  |  |  | Hopelessness | Right now how much do you feel: hopeless? | 0.78 |
|  | Clinical (outpatient) | 20 | Varied (up to 7 weeks) | 6 | Pain | Right now how much do you feel: physical pain? | 0.24 |
|  |  |  |  |  | Hopelessness | Right now how much do you feel: hopeless? | 0.45 |
| Kudinova et al., 2023 [65] | Clinical (inpatient) | 158 | 21 | 5 | Shame | Participants were asked to rate how they were feeling ‘right now’ (e.g., anger at self) | 0.11 |
|  |  |  |  |  | NSSI | Have you done anything to hurt your body on purpose, but without the intent to kill yourself? | n/a |
| Kuehn, 2022 [66] | Clinical (outpatient) and non-clinical | 60 | 14 | 5 | Coping (cognitive re-appraisal) | - | 0.31 |
|  |  |  |  |  | Coping (self-invalidation) |  | 0.32 |
|  |  |  |  |  | Coping (suppression) |  | 0.31 |
|  |  |  |  |  | Coping (distraction) |  | 0.24 |
|  |  |  |  |  | Coping (acceptance) |  | 0.25 |
|  |  |  |  |  | Coping (avoidance) |  | 0.04 |
|  |  |  |  |  | Rumination |  | 0.16 |
|  |  |  |  |  | Problem solving |  | 0.25 |
|  |  |  |  |  | Social support |  | 0.02 |
|  |  |  |  |  | Impulsivity |  | 0.75 |
|  |  |  |  |  | Shame |  | 0.38 |
|  |  |  |  |  | SITBs | Since the last assessment, have you thought about harming yourself? Participants who answered yes were then asked the strength of their intention of harming themselves in the past 30 minutes, the strength of their intention to kill themselves in the past 30 minutes, the current strength of their intention to kill themselves, and whether or not they engaged in any self-harm behavior in the past 30 minutes | n/a |
| López et al., 2023 [67] | University | 49 | 28 | 6 | Thwarted belongingness | Have you felt rejected by a friend? Have you felt excluded by a Group? Have you felt rejected by others? How excluded by other people have you felt? How alone have you felt? | 0.39 |
| Lucht et al., 2022 [35] | See Hallensleben et al., 2019 | | | | Impulsivity (1x per day) | - | 4 items ranging from 0.22-0.36 |
|  |  |  |  |  | Suicidal ideation | Passive suicidal ideation …life is not worth living for me. …there are more reasons to die than to live for me. Active suicidal ideation ...I want to die. ...I think about taking my life. | n/a |
| MacNeil et al., 2023 [68] | Clinical (outpatient) and non-clinical | 55 | 10 | 1 | Thwarted belongingness | Participants rated the extent to which they felt lonely today | 0.53 |
|  |  |  |  |  | Perceived burdensomeness | Participants selected the statement that applied best to them in the past 24 h from the following: 0 –I think that people in my life are happier when I'm around; (1) –I do not think that people in my life would be happier if I were gone; (2) –I wonder that people in my life would be happier if I were gone; (3) – I am sure that people in my life would be happier if I were gone. | 0.67 |
| Mitchell et al., 2023 [69] | University | 41 | 5 | 1 | Rumination | Please think back to the single most emotionally provoking negative event that happened to you today…” I thought about how I felt about what I had experienced; I was preoccupied with what I thought and felt about what I had experienced | 0.22 |
| Molaie, 2022 [70] | University | 197 | 14 | 1 | Thwarted belongingness | Rate how you feel right now: (1) I feel close to others. (2) I feel like I belong. | 0.67 |
| Mournet et al., 2022 [39] | University | 74 | 56 | 6 | Loneliness (1x per day) | How lonely do you feel right now? | - |
|  |  |  |  |  | Perceived burdensomeness (1x per day) | How burdensome did you feel today? | - |
|  |  |  |  |  | Suicidal ideation | - | n/a |
| Nuij et al., 2022 [26] | Clinical (outpatient) | 17 | 90 | 4 | Perceived burdensomeness | I’m a burden to others. | 0.86 |
|  |  |  |  |  | Thwarted belongingness (1x per day) | Today I felt close to other people. | - |
|  |  |  |  |  | Entrapment | I feel trapped | 0.52 |
|  |  |  |  |  | Future thoughts (1x per day) | I’m looking forward to tomorrow. | 0.6 |
|  |  |  |  |  | Hopelessness | - | 0.44 |
|  |  |  |  |  | Mental Imagery | During the past hour, mental images related to suicide came to mind. | 0.36 |
|  |  |  |  |  | Impulsivity (1x per day) | Today I acted without thinking. | 0.75 |
|  |  |  |  |  | Problem-solving/ coping (1x per day) | Today I felt capable of solving my problems. | 0.66 |
|  |  |  |  |  | Rumination | I wish today had gone better. | 0.47 |
| Parrish et al., 2021 [71] | Clinical (outpatient) | 96 | 10 | 3 | Perceived burdensomeness | How much have you felt that you were a burden on others? | 0.69 |
|  |  |  |  |  | Thwarted belongingness | How much have you been feeling like you belong or fit with others in your life? | 0.59 |
| Peters et al., 2022 [32] | Clinical (inpatient) | 39 | Varied (M=12) | 3 | Social connectedness (i.e., thwarted belongingness) | How close or connected to people do you feel right now? | 0.56 |
|  |  |  |  |  | Suicidality | How suicidal are you right now? | n/a |
| Reeves, 2022 [72] | Clinical (outpatient) | 10 | 14 | 9 | Thwarted belongingness | I feel like I fit in; I feel close to other people. | 0.74 |
|  |  |  |  |  | Perceived burdensomeness | I feel like a burden | 0.44 |
|  |  |  |  |  | Hopelessness | - | 0.48 |
| Rogers, 2023 [73] | Non-clinical | 237 | 14 | 6 | Rumination | I have been dwelling on my mistakes, failures, or losses”; “I keep thinking about something negative that has happened” | 0.56 |
|  |  |  |  |  | Suicide-Specific Rumination | - | 0.8 |
|  |  |  |  |  | Thwarted belongingness | I have felt like I belong’ I have felt lonely | 0.54 |
|  |  |  |  |  |  |  |  |
|  |  |  |  |  | Perceived burdensomeness | I have felt like a burden on the people in my life; I have felt useless | 0.63 |
|  |  |  |  |  | Hopelessness | - | 0.63 |
|  |  |  |  |  | Fearlessness about death | “I am not at all afraid to die”; “The pain involved in dying frightens me” | 0.88 |
|  |  |  |  |  | Access to means | Please indicate on the scale below how physically close you currently are to the primary suicide method you are considering | 0.66 |
|  |  |  |  |  | Suicidal ideation & intent | Suicide plans (“Since the last assessment, have you made or added to a plan to kill yourself?”), preparations (“Since the last assessment, have you made any preparations for a suicide attempt?”), and attempts (“Since the last assessment, have you made a suicide attempt?” | n/a |
| Selby et al., 2019 [74] | Clinical (outpatient) and non-clinical | 47 | 14 | 5 | Physical pain (event-contingent) – pre-NSSI pain rating | - | 0.26 |
|  |  |  |  |  | NSSI behaviours | Have you engaged in self-injury? | n/a |
| Silva et al., 2022 [75] | Clinical (outpatient) | 16 | 14 | 4 | Thwarted belongingness | Four items from INQ-S | 0.85 |
|  |  |  |  |  | Perceived burdensomeness | Five items from INQ-S | 0.81 |
|  |  |  |  |  | Emotional loneliness | DeJong Gierveld Loneliness Scale | 0.85 |
|  |  |  |  |  | Social loneliness |  | 0.96 |
|  |  |  |  |  | Suicidal ideation | Beck Scale for Suicide Ideation (BSS) | n/a |
| Spangenberg et al., 2019 [36] | See Hallensleben et al., 2019 | | | | Fearlessness about death (1x per day) | Today I was not at all afraid to die. | 0.47 |
|  |  |  |  |  | Pain tolerance (1x per day) | Today I would have taken a lot of (physical) pain | 0.52 |
| Stanley et al., 2021 [76] | Clinical (outpatient) | 50 | 7 | 6 | Coping strategies (engagement and effectiveness) | Participants reported whether or not they used each of the following seven strategies to cope since the last epoch: keeping busy; socializing; positive thinking; doing something good for self; calming self; finding perspective; and sitting with feelings until they passed. Participants rated the extent to which they considered the coping strategies they used to be effective in reducing distress | - |
|  |  |  |  |  | Suicidal ideation | Participants were asked to rate how strongly they experienced each of the following since the last epoch on a 5-point (0–4) Likert scale: a wish to live; a wish to die; a wish to escape; thoughts about dying; thoughts about suicide; urge to die by suicide; thoughts about hurting self; urges to hurt self; and whether they had reasons for living. | n/a |
| Stenzel et al., 2020 [77] | Non-clinical | 61 | 7 | 5 | Defeat | - | 0.52 |
|  |  |  |  |  | Entrapment | - | 0.54 |
| Tsypes et al., 2022 [37] | See Kaurin et al., 2023 | | | | Reasons for living (1x per day) | Participants were asked to what extent “Over the last 24 h” they thought a) that they can learn to adjust or cope with their problems; b) that they have control over their life and destiny; c) about their responsibilities to their family; d) about how much they love and enjoy their family; e) about future plans they look forward to carrying out; f) that everything has a way of working out for the best | 0.63 |
|  |  |  |  |  | Suicidal ideation | “Have you wished you were dead or wished you could go to sleep and not wake up?”, “Have you actually had any thoughts of killing yourself?” | n/a |
| Turner et al., 2016 [33] | Non-clinical | 60 | 14 | 1 | Perceived social support | 12 adjective pairs to assess perceptions of support(e.g., “My romantic partner was . . . helpful— unhelpful, selfish— generous). Participants provided ratings for three potential sources of support: romantic partners, parents, and peers. | 0.56 |
|  |  |  |  |  | NSSI urges | Alexian Brothers Urges to Self-Injure Scale | n/a |
| Turner et al., 2019 [40] | See Turner et al., 2016 | | | | Daily coping strategies | - | n/a |
|  |  |  |  |  | NSSI urges | Alexian Brothers Urges to Self-Injure Scale | n/a |
| van Ballegooijen et al., 2022 [78] | Clinical and non-clinical | 51 | 7 | 6 | Defeat | Right now, I feel emotionally defeated | 0.47 |
|  |  |  |  |  | Entrapment | Right now, I feel trapped; Right now, I want to escape my emptional pain | 0.58 |
|  |  |  |  |  | Suicidal ideation | Right now, I feel suicidal | n/a |
| Victor et al., 2019 [23] | Non-clinical | 63 | 21 | 6 | Interpersonal stress (thwarted belongingness) | Since the last prompt have you felt insulted or criticized? | 0.1 |
|  |  |  |  |  | Rejection (thwarted belongingness) | Since the last prompt have you felt rejected, abandoned, excluded, or left out? | 0.17 |
|  |  |  |  |  | Self-injurious and suicidal urges | Since the last prompt have you felt an urge or wanted to harm or injure yourself on purpose, without wanting to die (such as wanting to cut or burn yourself)? Since the last prompt have you felt the urge or wanted to make a suicide attempt? | n/a |
| Wolford‐Clevenger et al., 2020 [79] | University | 206 | 90 | 1 | Thwarted belongingness | “I felt close to others,” and “I felt like I belonged.” | - |
|  |  |  |  |  | Perceived burdensomeness | “I felt that the people in my life would have been happier without me,” and “I felt that the people in my life would have been better off if I were gone.” | - |
|  |  |  |  |  | Hopelessness | Profile of Mood States, Short Form (POMS-SF) | - |
|  |  |  |  |  | capability for suicide (pain tolerance and fearlessness about death) | - | - |
|  |  |  |  |  | Suicidal ideation | Paykel Suicide Scale (PSS) | n/a |
| Wolford‐Clevenger et al., 2021 [28] | Non-clinical (including University) | 38 | 30 | 1 | Hopelessness | Participants ranked how “hopeless” they felt the day prior on a scale of 0 = “Not at all” to 4 = “Extremely”. | - |
|  |  |  |  |  | Social connectedness (thwarted belongingness) | “I felt close to others” and “I felt like I belonged” | - |
|  |  |  |  |  | Suicidal ideation | Paykel Suicide Scale (PSS) | n/a |

References

1. Cloos M, Di Simplicio M, Hammerle F, Steil R. Mental images, entrapment and affect in young adults meeting criteria of nonsuicidal self-injury disorder (NSSID) - a daily diary study. Borderline Personality Disorder and Emotion Dysregulation. 2020 ;7:4. DOI: 10.1186/s40479-019-0117-0. PMID: 32071721; PMCID: PMC7014591.
2. Victor SE, Scott LN, Stepp SD, Goldstein TR. I want you to want me: Interpersonal stress and affective experiences as within‐person predictors of nonsuicidal self‐injury and suicide urges in daily life. Suicide and Life‐Threatening Behavior. 2019 Aug;49(4):1157-77. PMID: 30159910
3. Wolford‐Clevenger C, Flores LY, Stuart GL. Proximal correlates of suicidal ideation among transgender and gender diverse people: A preliminary test of the three‐step theory. Suicide and Life‐Threatening Behavior. 2021 Dec;51(6):1077-85. PMID: 34254694
4. Baryshnikov I, Rosenström T, Isometsä E. Predicting a short-term change of suicidal ideation in inpatients with depression: An ecological momentary assessment. Journal of affective disorders. 2024 Apr 1;350:1-6. PMID: 38232774
5. Bentley KH, Coppersmith DL, Kleiman EM, Nook EC, Mair P, Millner AJ, Reid-Russell A, Wang SB, Fortgang RG, Stein MB, Beck S. Do patterns and types of negative affect during hospitalization predict short-term post-discharge suicidal thoughts and behaviors?. Affective science. 2021 Dec;2:484-94. PMID: 35465415
6. Kellerman JK, Millner AJ, Joyce VW, Nash CC, Buonopane R, Nock MK, Kleiman EM. Social support and nonsuicidal self-injury among adolescent psychiatric inpatients. Research on child and adolescent psychopathology. 2022 Oct;50(10):1351-61. PMID: 35579780
7. Kleiman EM, Turner BJ, Fedor S, Beale EE, Huffman JC, Nock MK. Examination of real-time fluctuations in suicidal ideation and its risk factors: Results from two ecological momentary assessment studies. Journal of abnormal psychology. 2017 Aug;126(6):726. PMID: 28481571
8. Krall HR, Ruork AK, Rizvi SL, Kleiman EM. Hopelessness as a Mechanism of the Relationship between Physical Pain and Thoughts of Suicide: Results from Two Smartphone-Based Real-Time Monitoring Samples. Cognitive Therapy and Research. 2024 Mar 4:1-8.
9. Peters EM, Dong LY, Thomas T, Khalaj S, Balbuena L, Baetz M, Osgood N, Bowen R. Instability of suicidal ideation in patients hospitalized for depression: an exploratory study using smartphone ecological momentary assessment. Archives of suicide research. 2022 Jan 2;26(1):56-69. PMID: 32654657
10. Turner BJ, Cobb RJ, Gratz KL, Chapman AL. The role of interpersonal conflict and perceived social support in nonsuicidal self-injury in daily life. Journal of abnormal psychology. 2016 May;125(4):588. PMID: 26845256
11. Turner BJ, Baglole JS, Chapman AL, Gratz KL. Experiencing and resisting nonsuicidal self‐injury thoughts and urges in everyday life. Suicide and Life‐Threatening Behavior. 2019 Oct;49(5):1332-46. PMID: 30152181
12. Al-Dajani N, Czyz EK. Suicidal desire in adolescents: an examination of the interpersonal psychological theory using daily diaries. Journal of Clinical Child & Adolescent Psychology. 2022 Apr 22:1-5. PMID: 35476615
13. Czyz EK, Koo HJ, Al-Dajani N, King CA, Nahum-Shani I. Predicting short-term suicidal thoughts in adolescents using machine learning: developing decision tools to identify daily level risk after hospitalization. Psychological medicine. 2023 May;53(7):2982-91. PMID: 34879890
14. Al‐Dajani N, Horwitz AG, Czyz EK. Does coping reduce suicidal urges in everyday life? Evidence from a daily diary study of adolescent inpatients. Depression and anxiety. 2022 Jun;39(6):496-503. PMID: 35322919
15. Czyz EK, Horwitz AG, Arango A, King CA. Short‐term change and prediction of suicidal ideation among adolescents: A daily diary study following psychiatric hospitalization. Journal of child psychology and psychiatry. 2019 Jul;60(7):732-41. PMID: 30246870
16. Czyz EK, Glenn CR, Busby D, King CA. Daily patterns in nonsuicidal self-injury and coping among recently hospitalized youth at risk for suicide. Psychiatry research. 2019 Nov 1;281:112588. <https://doi.org/10.1016/j.psychres.2019.112588>. PMID: 31629299
17. Lucht L, Hallensleben N, Willhardt N, Forkmann T, Rath D, Glaesmer H, Spangenberg L. Daily impulsivity: Associations with suicidal ideation in unipolar depressive psychiatric inpatients. Psychiatry research. 2022;308: 114357. PMID: 34974411
18. Hallensleben N, Glaesmer H, Forkmann T, Rath D, Strauss M, Kersting A, Spangenberg L. Predicting suicidal ideation by interpersonal variables, hopelessness and depression in real-time. An ecological momentary assessment study in psychiatric inpatients with depression. European psychiatry. 2019 Feb;56(1):43-50. <http://doi.org/10.1016/j.eurpsy.2018.11.003>. PMID: 30530103
19. Spangenberg L, Glaesmer H, Hallensleben N, Rath D, Forkmann T. (In) stability of capability for suicide in psychiatric inpatients: longitudinal assessment using ecological momentary assessments. Suicide and Life‐Threatening Behavior. 2019 Dec;49(6):1560-72. PMID: 30834576
20. Kaurin A, Dombrovski AY, Hallquist MN, Wright AG. Suicidal urges and attempted suicide at multiple time scales in borderline personality disorder. Journal of affective disorders. 2023 May 15;329:581-8. PMID: 36781143
21. Tsypes A, Kaurin A, Wright AG, Hallquist MN, Dombrovski AY. Protective effects of reasons for living against suicidal ideation in daily life. Journal of psychiatric research. 2022 Apr 1;148:174-80. PMID: 35124397
22. Kirtley OJ, Lafit G, Vaessen T, Decoster J, Derom C, Gülöksüz S, De Hert M, Jacobs N, Menne-Lothmann C, Rutten BP, Thiery E, van Os J, van Winkel R, Wichers M, Myin-Germeys I The relationship between daily positive future thinking and past-week suicidal ideation in youth: An experience sampling study. Frontiers in Psychiatry. 2022 Sep 29;13:915007. PMID: 36245862
23. Mournet AM, Kellerman JK, Yeager AL, Rosen RL, Kim JS, Kleiman EM. Daily‐level assessment of the contexts under which seeking social support relates to risk of suicidal thinking. Suicide and Life‐Threatening Behavior. 2022 Dec;52(6):1159-67. PMID: 35972392
24. Nuij C, van Ballegooijen W, de Beurs D, de Winter RF, Gilissen R, O’Connor RC, Smit JH, Kerkhof A, Riper H. The feasibility of using smartphone apps as treatment components for depressed suicidal outpatients. Frontiers in psychiatry. 2022 Sep 27;13:971046. PMID: 36238944
25. Aadahl V, Wells A, Hallard R, Pratt D. Metacognitive beliefs and suicidal ideation: an experience sampling study. International journal of environmental research and public health. 2021 Nov 24;18(23):12336. PMID: 34886060
26. Al-Dajani N, Uliaszek AA. The after-effects of momentary suicidal ideation: A preliminary examination of emotion intensity changes following suicidal thoughts. Psychiatry research. 2021 Aug 1;302:114027. PMID: 34139594
27. Ammerman BA, Olino TM, Coccaro EF, McCloskey MS. Predicting nonsuicidal self-injury in borderline personality disorder using ecological momentary assessment. Journal of personality disorders. 2017 Dec;31(6):844-55. PMID: 28072044
28. Bayliss LT, Hughes CD, Lamont‐Mills A, du Plessis C. Fluidity in capability: Longitudinal assessments of suicide capability using ecological momentary assessments. Suicide and Life‐Threatening Behavior. 2024 Feb;54(1):138-53. PMID: 38009897
29. Ben-Zeev D, Young MA, Depp CA. Real-time predictors of suicidal ideation: mobile assessment of hospitalized depressed patients. Psychiatry research. 2012 May 15;197(1-2):55-9. PMID: 22397912
30. Burke TA, Allen KJ, Carpenter RW, Siegel DM, Kautz MM, Liu RT, Alloy LB. Emotional response inhibition to self-harm stimuli interacts with momentary negative affect to predict nonsuicidal self-injury urges. Behaviour research and therapy. 2021 Jul 1;142:103865. PMID: 33940222
31. Christensen K, Victor SE, Littlefield AK, Mitchell SM. A comparison of retrospectively reported and ecological momentary assessment‐reported perceived social support in predicting ecological momentary assessment‐reported non‐suicidal self‐injury. Suicide and Life‐Threatening Behavior. 2024 Apr;54(2):184-94. PMID: 38078550
32. Coppersmith DD, Kleiman EM, Glenn CR, Millner AJ, Nock MK. The dynamics of social support among suicide attempters: A smartphone-based daily diary study. Behaviour research and therapy. 2019 Sep 1;120:103348. PMID: 30594300
33. Czyz EK, King CA, Al-Dajani N, Zimmermann L, Hong V, Nahum-Shani I. Ecological Momentary Assessments and Passive Sensing in the Prediction of Short-Term Suicidal Ideation in Young Adults. JAMA Network Open. 2023 Aug 1;6(8):e2328005-. PMID: 37552477
34. Defayette AB, Esposito‐Smythers C, Cero I, Kleiman EM, López Jr R, Harris KM, Whitmyre ED. Examination of proinflammatory activity as a moderator of the relation between momentary interpersonal stress and suicidal ideation. Suicide and Life‐Threatening Behavior. 2023 Dec;53(6):922-39. PMID: 37578098
35. Ewing L, Hamza CA. A Diary Study of the Within-Person Associations Between Daily Stressors and Negative Affect Among Post-Secondary Students With Recent Nonsuicidal Self-Injury Engagement. Emerging Adulthood. 2024 Mar 18:21676968241239374.
36. Gerner JL, Moscardini EH, Mitchell SM, Hill RM, Tucker RP. Examination of real‐time variation in interpersonal hopelessness and suicidal desire in a college student sample reporting past‐2‐week suicidal ideation. Suicide and Life‐Threatening Behavior. 2023 Oct;53(5):893-905. PMID: 37578159
37. Glenn CR, Kleiman EM, Kandlur R, Esposito EC, Liu RT. Thwarted belongingness mediates interpersonal stress and suicidal thoughts: An intensive longitudinal study with high-risk adolescents. Journal of Clinical Child & Adolescent Psychology. 2022 May 4;51(3):295-311. PMID: 34570668
38. Hallard RI, Wells A, Aadahl V, Emsley R, Pratt D. Metacognition, rumination and suicidal ideation: An experience sampling test of the self-regulatory executive function model. Psychiatry Research. 2021 Sep 1;303:114083. PMID: 34271370
39. Harper KL. Interpersonal Perfectionism, Attributions, Expectations, and Social Disconnection in Daily Life: An Extension of the Social Disconnection Model. The University of North Carolina at Greensboro; 2019. Retrieved from https://bris.idm.oclc.org/login?url=https://www.proquest.com/dissertations-theses/interpersonal-perfectionism-attributions/docview/2301551348/se-2
40. Hughes CD, King AM, Kranzler A, Fehling K, Miller A, Lindqvist J, Selby EA. Anxious and overwhelming affects and repetitive negative thinking as ecological predictors of self-injurious thoughts and behaviors. Cognitive Therapy and Research. 2019 Feb 15;43:88-101.
41. Jacobucci R, McClure K, Ammerman BA. Comparing the role of perceived burdensomeness and thwarted belongingness in prospectively predicting active suicidal ideation. Suicide and Life‐Threatening Behavior. 2023 Apr;53(2):198-206. PMID: 36458583
42. Jeong S, An J, Cho S. Role of affective instability on suicidal risk in complex regional pain syndrome: a diary approach (preliminary report). The Korean journal of pain. 2021 Jan 1;34(1):94. PMID: 33380572
43. Kaurin A, Dombrovski AY, Hallquist MN, Wright AG. Momentary interpersonal processes of suicidal surges in borderline personality disorder. Psychological medicine. 2022 Oct;52(13):2702-12. PMID: 33298227
44. Kudinova AY, Brick LA, Armey M, Nugent NR. Micro‐sequences of anger and shame and non‐suicidal self‐injury in youth: An ecological momentary assessment study. Journal of child psychology and psychiatry. 2024 Feb;65(2):137-47. PMID: 37525367
45. Kuehn KS. Using Bayesian mixed-effects models to predict self-injurious thoughts in intensive longitudinal data. University of Washington; 2022.
46. López Jr R, Esposito-Smythers C, Defayette AB, Harris KM, Seibel LF, Whitmyre ED. Facets of social problem-solving as moderators of the real-time relation between social rejection and negative affect in an at-risk sample. Behaviour Research and Therapy. 2023 Oct 1;169:104398. PMID: 37708724
47. MacNeil S, Renaud J, Gouin JP. Respiratory sinus arrhythmia, negative social interactions, and fluctuations in unmet interpersonal needs: A daily diary study. Suicide and Life‐Threatening Behavior. 2023 Aug;53(4):597-612. PMID: 37208985
48. Mitchell E, Rosario-Williams B, Yeshchenko I, Miranda R. Cognitive emotion regulation strategies among emerging adults with different self-harm histories. Journal of affective disorders reports. 2023 Dec 1;14:100638. PMID: 38047209
49. Molaie AM. The Context of Loneliness in Young Adulthood: An Exploratory Examination of the Construct of Thwarted Belongingness (Doctoral dissertation, University of Nevada, Reno).
50. Parrish EM, Chalker SA, Cano M, Moore RC, Pinkham AE, Harvey PD, Joiner T, Lieberman A, Granholm E, Depp CA. Ecological momentary assessment of interpersonal theory of suicide constructs in people experiencing psychotic symptoms. Journal of psychiatric research. 2021 Aug 1;140:496-503. PMID: 34157588
51. Reeves K. Within-Person Patterns of Adolescent Suicidal Ideation and Related Risk Factors. University of California, San Francisco; 2022.
52. Rogers ML. A real-time evaluation of within-person and between-person risk for suicidal behaviors. International Journal of Cognitive Therapy. 2024 Mar;17(1):72-92.
53. Selby EA, Kranzler A, Lindqvist J, Fehling KB, Brillante J, Yuan F, Gao X, Miller AL. The dynamics of pain during nonsuicidal self-injury. Clinical Psychological Science. 2019 Mar;7(2):302-20
54. Silva C, Cero I, Ricci N, Pérez A, Conwell Y, Van Orden K. The feasibility and acceptability of using smartphones to assess suicide risk among Spanish‐speaking adult outpatients. Suicide and Life‐Threatening Behavior. 2022 Oct;52(5):918-31. PMID: 35674249
55. Stanley B, Martínez-Alés G, Gratch I, Rizk M, Galfalvy H, Choo TH, Mann JJ. Coping strategies that reduce suicidal ideation: An ecological momentary assessment study. Journal of psychiatric research. 2021 Jan 1;133:32-7. PMID: 33307352
56. Stenzel JS, Höller I, Rath D, Hallensleben N, Spangenberg L, Glaesmer H, Forkmann T. Do feelings of defeat and entrapment change over time? An investigation of the integrated motivational—Volitional model of suicidal behaviour using ecological momentary assessments. International journal of environmental research and public health. 2020 Jul;17(13):4685. PMID: 32610667
57. van Ballegooijen W, Littlewood DL, Nielsen E, Kapur N, Gooding P. The temporal relationships between defeat, entrapment and suicidal ideation: ecological momentary assessment study. BJPsych open. 2022 Jul;8(4):e105. PMID: 35656578
58. Wolford‐Clevenger C, Stuart GL, Elledge LC, McNulty JK, Spirito A. Proximal correlates of suicidal ideation and behaviors: A test of the interpersonal‐psychological theory of suicide. Suicide and Life‐Threatening Behavior. 2020 Feb;50(1):249-62. PMID: 31503359
